# Graph neural network-based risk stratification of prostate cancer using gene expression and SHAP interpretability

**DOI:** 10.1371/journal.pone.0356727

**Published:** 2026-09-08

**Authors:** Saeed Pirmoradi, Sevil Vaghefi Moghaddam, Mohammadreza Ardalan, Mir Mohsen Sharifi Bonab, Mohammad Teshnehlab, Sepideh Zununi Vahed

**Affiliations:** 1 Neurosciences Research Center, Tabriz University of Medical Sciences, Tabriz, Iran; 2 Faculty of Electronic and Computer Engineering Department, K.N. Toosi University of Technology, Tehran, Iran; 3 Clinical Research Development Unit of Tabriz Valiasr Hospital, Tabriz University of Medical Sciences, Tabriz, Iran; 4 Kidney Research Center, Tabriz University of Medical Sciences, Tabriz, Iran; Jan Biziel University Hospital No 2 in Bydgoszcz: Szpital Uniwersytecki Nr 2 im dr Jana Biziela w Bydgoszczy, POLAND

## Abstract

Accurate risk stratification is essential for guiding treatment decisions and preventing over treatment of prostate cancer, which remains one of the most prevalent cancers among adult men. While the Gleason score, obtained from prostate biopsies, is routinely used to assess tumor aggressiveness, the biopsy procedure carries risks such as pain, infection, and, in some cases, serious complications such as sepsis. In this study, we proposed an artificial intelligence-based framework that integrates mRNA expression profiles with functional interaction networks to classify prostate cancer patients into low-, medium-, and high-risk groups defined by Gleason scores. The pipeline comprised five steps: (1) data collection from The Cancer Genome Atlas (TCGA), (2) preprocessing of gene expression data, (3) two-stage feature selection to identify informative biomarkers, (4) risk classification using a dual-branch graph neural network (GNN) that combines gene-gene interaction graphs with sample-level expression features, and (5) model interpretation using SHAP to quantify feature contributions. Differentially expressed genes were identified in the High (ASPN, GMNN, PEBP4, C2, KNCK17), Medium (C2, IGSF1, ASPN, CDKN3, AMH), and Low (TNMD, VWA5B2, ST6GALNAC5, CYP3A5, PHGR1) risk groups, underscoring the molecular heterogeneity of disease progression. On an independent held-out test set, the model achieved AUCs of 0.86, 0.88, and 0.95 for the low-, medium-, and high-risk groups, respectively, with an overall accuracy of 80%. These results suggest that combining GNN-based modeling with explainable AI can capture both global and local molecular patterns relevant to tumor aggressiveness. However, as the model was developed and evaluated solely on the TCGA cohort, the findings should be regarded as exploratory, and external validation will be required to establish generalizability. Within these limitations, the proposed framework highlights the potential of molecular profiling and graph-based deep learning to support more precise, potentially less invasive, risk assessment and individualized treatment planning in prostate cancer.

## Introduction

In 2024, prostate cancer (PCa) is projected to be the most prevalent cancer among men in the US, with an estimated 299,010 new cases diagnosed. It makes up 14.9% of all new cancer cases in both genders and 29% of all newly diagnosed cancers in men [1]. Identifying biomarkers that differentiate benign from malignant tumors has been the focus of numerous studies. Meanwhile, specific clinical characteristics of malignant tumors, including invasiveness, survival, progression, and recurrence, have received increased attention from researchers in recent years.

Risk stratification, or categorizing patients into high-risk (tumors that grow quickly) and low-risk (tumors that grow slowly) groups, is essential in prostate cancer. Early detection of high-risk patients can help prevent overtreatment [2] and significantly increase survival rates [3]. The TNM system, developed by Pierre Denoix in 1950, is one of the most reliable staging systems for solid tumors and is used for risk assessment. Currently, this system is widely utilized for treating various cancers [4]. According to the patient’s pathological imaging, TNM represents the size and extent of the primary tumor, N indicates lymph node involvement, and M denotes distant metastases [5].

The Gleason score, which characterizes the overall architecture of tissue and indicates the aggressiveness of the disease, is another essential score in prostate cancer determined using these metrics [6]. Based on the degree of aggressiveness and invasiveness of the tumor, a pathologist assigns a Gleason score, which is the sum of two numbers, each ranging from 3 to 5. As shown in Table 1, risk groups are categorized using the Gleason score, which ranges from 1 to 5.

**Table 1:** Risk groups based on the Gleason score [7].

Grade Group	Gleason Score	Combined Gleason Score	Aggressiveness degree
1	3+3	6	Low risk
2	3+4	7	Intermediate risk but closer to low risk
3	4+3	7	Intermediate risk but closer to high risk
4	4+4, 3+5, 5+3	8	High risk
5	4+5, 5+4, 5+5	9, 10	High risk

The Prostate-Specific Antigen (PSA) test and Digital Rectal Examination (DRE) are part of the European Association of Urology’s (EAU) guidelines, regarded as some of the most reliable methods for identifying suspected prostate cancer. The sensitivity and specificity of these two diagnostic procedures are both low. Therefore, if either test produces a positive result, the individual is referred to a radiologist for a magnetic resonance imaging (MRI) scan. Ultimately, a prostate biopsy conducted by a qualified urologist or an experienced radiologist is essential to ascertain the patient’s or suspected individual’s risk level for prostate cancer. A pathologist subsequently analyzes the biopsy sample to evaluate the risk of cancer.

Patients undergoing prostate biopsies may face several challenges despite this method being regarded as an accurate diagnostic tool for detecting prostate cancer and evaluating its risk level. These challenges include pain, the invasive nature of the biopsy procedure, a significant risk of post-procedure infections, and the potential for sepsis, which affects 2–3% of patients and can lead to organ failure and death [8].

These days, machine learning techniques have emerged as a reliable method for analyzing various types of genetic data, particularly in interpreting mRNA expression profiles associated with different diseases. The following summarizes several recent studies conducted on prostate cancer. To identify biomarkers that could predict the progression of prostate cancer tumors toward more advanced stages, Alkhateeb et al. used supervised machine-learning techniques [9]. Arvaniti et al. utilized deep learning techniques to determine the Gleason score in a separate study [10]. They trained a deep-learning model using pathology images from 641 patients and validated it with images from an additional 245 patients. No gene-based biomarker diagnostic panel was available for predicting the Gleason score despite a reported accuracy of 85.72%. Similarly, Citak et al. used machine learning techniques, including support vector machines, to predict the Gleason score with 76.83% accuracy, utilizing prostate imaging data from a small population [11].

Furthermore, Osama Hamzeh et al. analyzed gene expression data from patients with prostate cancer, comparing each risk group at each stage pairwise with every other group [12]. Alkhateeb et al. reviewed recent developments in machine learning methods for identifying biomarkers related to critical clinical features of prostate cancer, including tumor laterality, Gleason score, and disease progression. With accuracies exceeding 90% across all tasks, supervised learning models trained on gene expression and next-generation sequencing datasets displayed impressive performance. Through the analysis, numerous significant biomarkers were identified, such as FBXO21, SNAI2, and HLA-DMB for predicting tumor laterality; CARNA22, DOCK9, and FLVCR2 for cancer progression; and UBE2V2 and GPR137 for Gleason score classification [13].

Considering the high incidence of prostate cancer and the risks associated with biopsy, this study addressed the urgent need for a non-invasive method to assess risk. Identifying key biomarkers related to risk groups defined by the Gleason score emphasizes the potential for directly predicting tumor aggressiveness from patients’ genetic profiles. The study underscored the clinical significance of the Gleason score in predicting cancer progression and recurrence. It further underscored the significance of deep learning and advanced machine learning techniques in developing an effective classification model and elucidating the molecular mechanisms of prostate cancer.

## Materials and Methods

### Materials

Data on genetic and clinical factors for prostate cancer patients were obtained from the Linked-Omics database (https://www.linkedomics.org/login.php) [14], which is provided by The Cancer Genome Atlas (TCGA) and affiliated with the National Institutes of Health (NIH) in the United States with TCGA-PRAD name (https://portal.gdc.cancer.gov/projects/TCGA-PRAD). The genetic data include gene expression levels in prostate cancer patients, covering 20,051 genes reported for 497 patients. Furthermore, clinical data were retrieved from the TCGA database, encompassing various clinical attributes, such as the Gleason score assigned to each patient by a specialized pathologist. In this study, patient classification was based on the “risk group” column, as detailed in Table 2. Detailed clinical statistics of the patients are presented separately in Table 3.

**Table 2:** Patients’ classification based on the Gleason score.

Gleason Score 1^st^	Gleason Score 2^nd^	Gleason Score	Risk Group	Class
3+3		6	Low (L)	1
3+4		7	Medium (M)	2
4+3		7	High (H)	3
4+4		8		
4+5, 5+5, 5+4		9, 10		

**Table 3:** Statistical information on PCa patients.

**• Gleason Group:** G1: 45 G2: 149 G3: 101 G4: 64 G5: 141
**• Pathologic N:** N0: 348 N1: 79 **• Pathologic T:** T2: 188 T3: 29 T4: 10 Not reported: 7
**• Laterality:** Left: 19 Right: 38 Bilateral: 435 Not reported: 8
**• History of other malignancies:** No: 471 Yes: 12 Yes, History of Prior Malignancy: 16 Yes, History of Synchronous/Bilateral Malignancy: 1
**• History of neoadjuvant treatment:** No: 498 Yes, Pharmaceutical Treatment Prior to Resection: 2
**• Residual tumor:** R0: 318 R1: 147 R2: 5 Rx: 15 Not reported: 15
**• Vital status:** Alive: 492 Dead: 8
**• Race:** American and Indian of Alaska native: 1 Black or African American: 58 Asian: 12 White: 415 Not reported: 14
**• Initial pathologic diagnosis method:** Core needle biopsy: 480 Transurethral resection (TURBT): 15 Not reported: 5
**• Age:** Mean: 61 Standard deviation: 6.8 Minimum: 41 Maximum: 78

See Supplementary S1 (section a) Files.

### Methods

**Fig 1:**
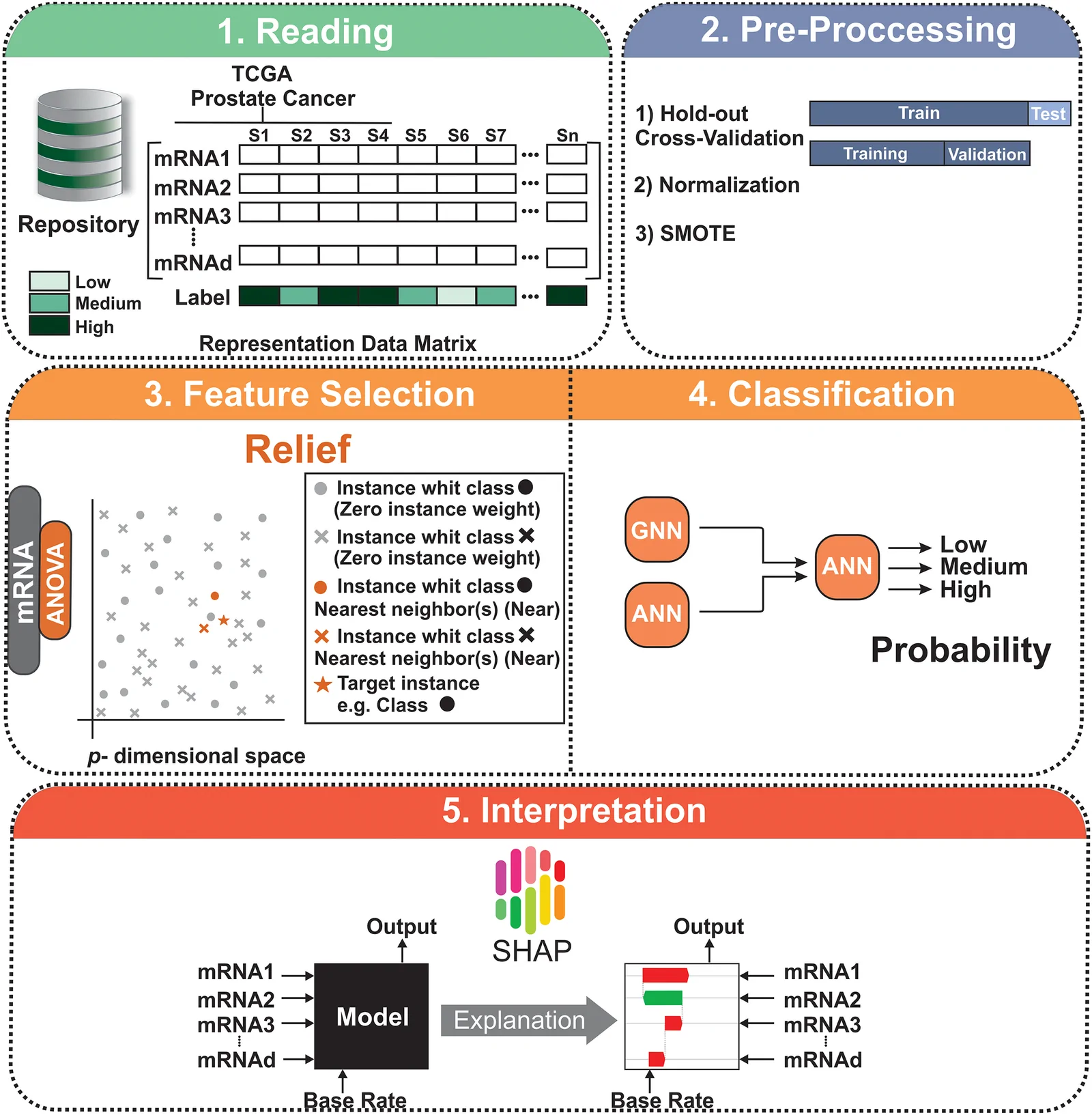
The proposed framework.

As shown in Fig 1, the proposed method comprises five steps, which are detailed in the following sections.

### First step: Reading

In this step, genetic and clinical data for prostate cancer patients were obtained from the Cancer Genome Atlas database. Approximately 20,000 gene expression values are reported for each patient, with a total of 497 patients included in the dataset. Additionally, the clinical data consists of the Gleason score for each patient, which is retrieved from the same database. Also, patients were classified according to the “risk group” column (Low, Medium, and High), as shown in Table 2. At the end of this step, a data matrix was prepared for the gene expression data, consisting of 20,051 rows and 497 columns. The Python programming environment, Jupyter Lab, and the libraries OS, Pickle, Pandas, and NumPy were used.

### Second step: Preprocessing

In this step, we first applied cross-validation to estimate the model’s error in real-world scenarios accurately [15]. The data were randomly divided into three groups: training, validation, and test sets, using the Hold-out method with proportions of 70%, 10%, and 20%, respectively. The number of data points in each group is shown in Table 4. The data matrix for each group was arranged so that the number of rows corresponds to the features, while the number of columns corresponds to the samples or patients in each group.

**Table 4:** Information on training, validation, and test sets.

Groups	Train			Validation			Test		
#	357			40			100		
**Risk Groups**	**L (0)**	**M (1)**	**H (2)**	**H(2)**	**M (1)**	**L (0)**	**L (0)**	**M (1)**	**H (2)**
#	32	105	220	24	12	4	9	29	62

L: low, M: medium, and H: high risk.

First, the missing values in the mRNA expression dataset were imputed using appropriate methods based on the training set. If the proportion of missing values for a given feature exceeded 10% of the total number of samples, that feature was excluded from the dataset. In the dataset used in this study, the TCGA mRNA expression matrix did not contain missing values; therefore, no imputation procedure was required. Additionally, three features in the gene expression data were removed from the training set due to the lack of variation in their expression values (the expression values contained constant value), resulting in a reduction of the feature count from 20,051–20,048.

Next, we utilized z-score scaling for feature normalization according to the requirements of subsequent steps. All data was normalized based on the information from the training set (mean and standard deviation) using the formula in equation (1), ensuring that the values fall within the range of [−1, 1].


$$\begin{array}{c} y=\;\frac{x-\mu }{\sigma } \end{array}$$ (1)

In equation (1), the variables x and y represent the input and normalized variables, respectively, while μ and σ denote the mean and standard deviation of the feature across all samples.

**Fig 2:**
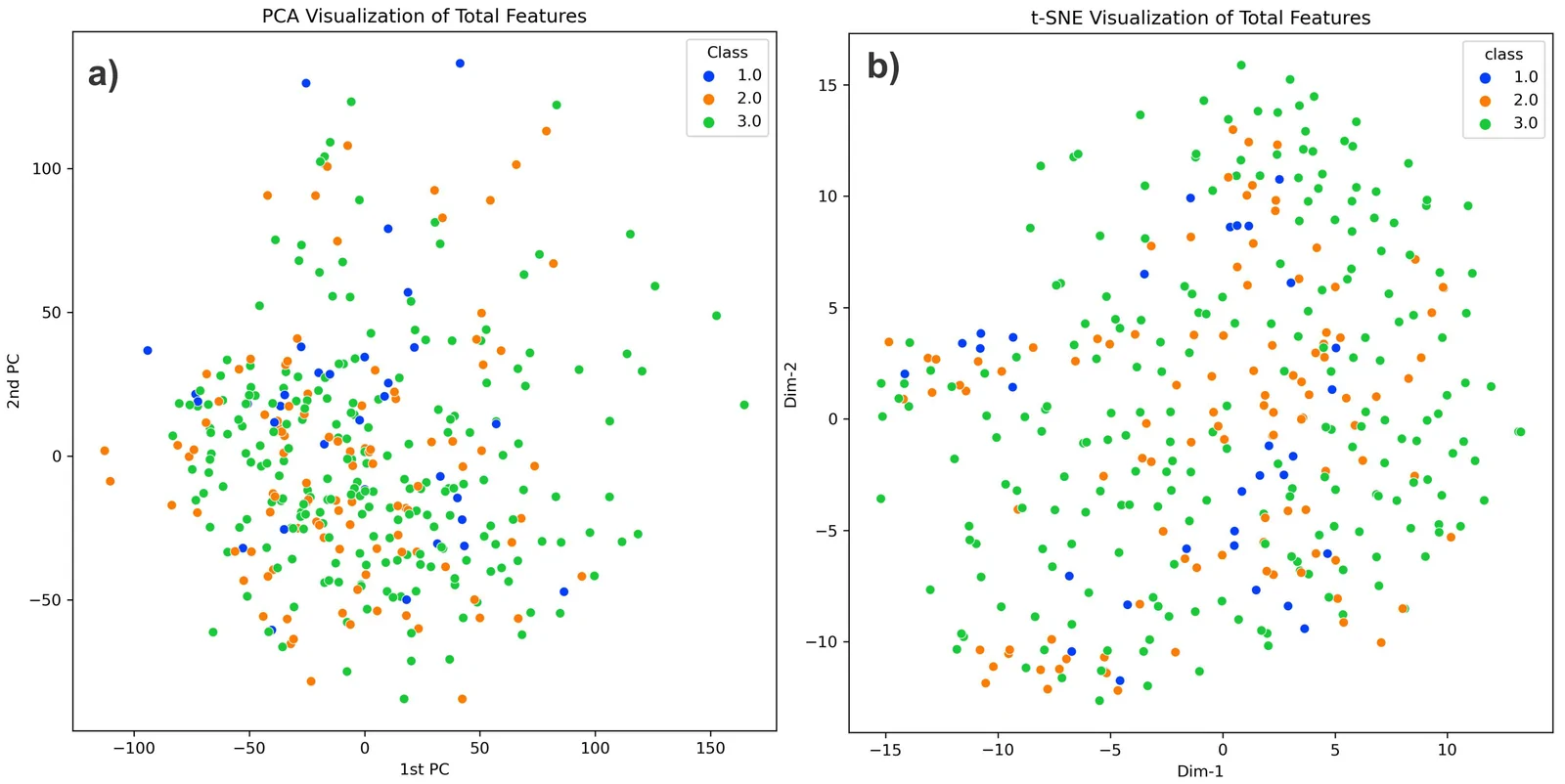
Visualizing the training set for all features (20,051 features) using two dimensions of (a) the PCA method, (b) the t-SNE method. In the figures, 0 class: low risk group, 1 class: medium risk group, 2 class: high risk group. See Supplementary S1 File (section b).

Initially, visualization methods based on Principal Components Analysis (PCA) and t-distributed Stochastic Neighbor Embedding (t-SNE) were used to examine the separability of risk groups in prostate cancer patients. Fig 2 visualizes the risk groups of prostate cancer patients in two dimensions using PCA and t-SNE methods, utilizing the training set while considering all features.

**Fig 3:**
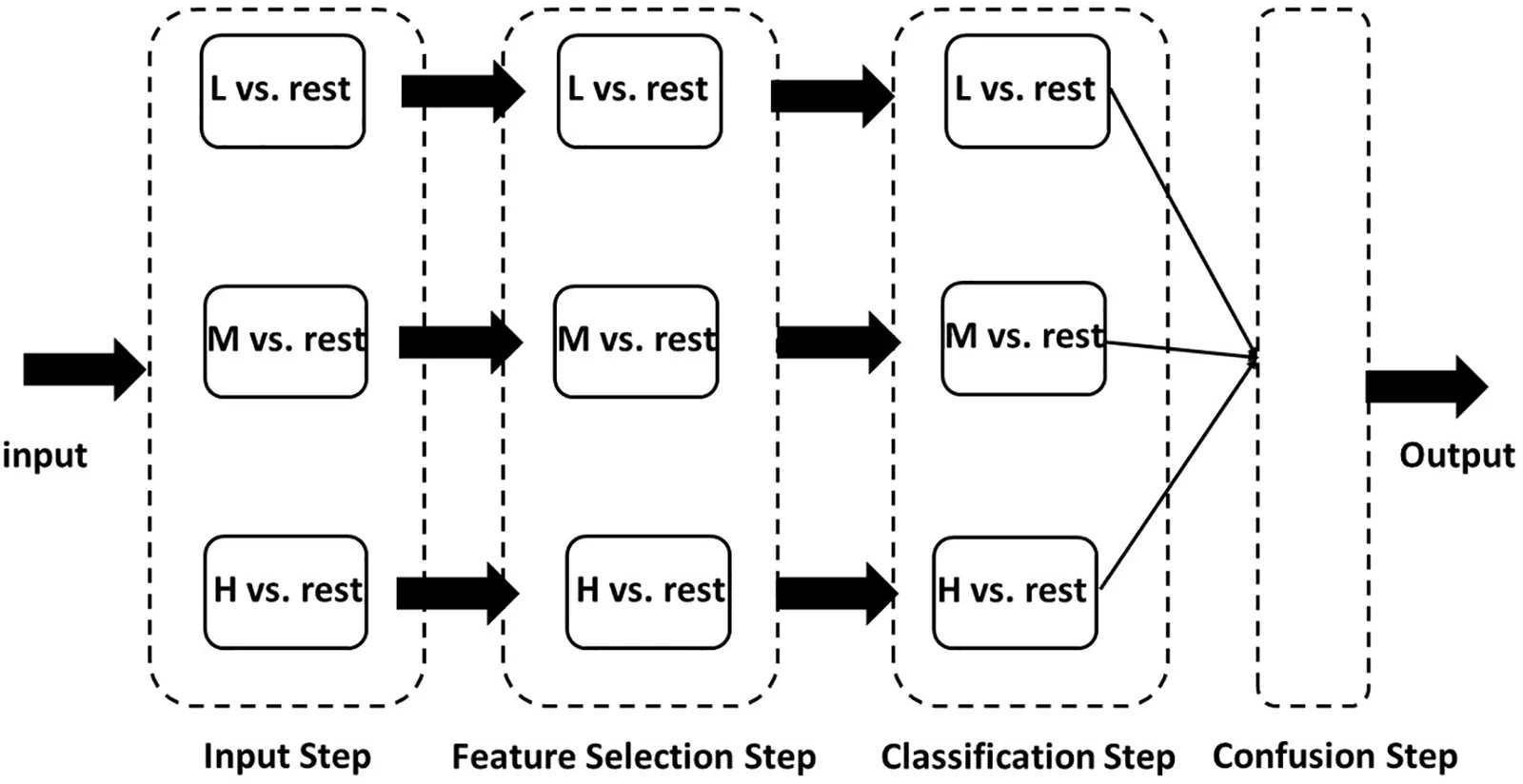
The proposed framework is based on the one vs. the rest strategy.

The generated plots clearly illustrate the complexity of the problem regarding the relationship between gene expression levels and risk subgroups. The challenge associated with multi-class classification (more than two classes) indicated that a one vs. rest strategy should be employed in both the feature selection and classification steps by utilizing an ensemble method that combines classifier results. The proposed framework, based on the one vs. rest strategy, is presented in Fig 3. Consequently, as indicated in Table 5, the training, validation, and test sets have been redefined into three groups according to the one vs. rest strategy. The details of the new data matrices, along with the count for each new class, are reported in Table 5. It is noted that all groups exhibit imbalanced new classes, and to advance to subsequent steps (feature selection and classification), it is essential to employ Over-sampling or Down-sampling methods. In this study, the oversampling method, specifically the SMOTE technique [16], was applied to the training data matrix of each group. The results of using the SMOTE method are detailed in Table 5. This process utilized the OS, Pickle, Scikit-learn, Imbalanced-learn, and NumPy libraries.

**Table 5:** The information about the training, validation, and test sets before and after the SMOTE oversampling method.

Defined Groups		Before SMOTE						After SMOTE					
		Train		Validation		Test		Train		Validation		Test	
		0	1	0	1	0	1	0	1	0	1	0	1
Group 1	L (0) vs. rest (1)	32	325	4	36	9	91	325	325	4	36	9	91
Group 2	M (0) vs. rest (1)	105	252	12	28	29	71	252	252	12	28	29	71
Group 3	H (0) vs. rest (1)	220	137	24	16	62	38	220	220	24	16	62	38

See Supplementary S1 (section b) Files.

L: low, M: medium, and H: high risk.

### Third step: Feature selection

The third step aimed to reduce the dimensionality of the gene expression data and identify the most informative genes for distinguishing among prostate cancer risk groups. Because the dataset is high‑dimensional, feature selection was performed in two sequential phases: (1) a filter‑based phase to remove irrelevant features based on statistical method, and (2) a supervised selection phase to refine the remaining features based on neighbors’ information. These steps were applied independently for each one‑vs‑rest comparison (High vs. Rest, Medium vs. Rest, and Low vs. Rest), consistent with the workflow shown in Fig 3.

#### Step 1: Filter‑based feature pre‑selection based on statistics.

1. Objective: Rapidly eliminate irrelevant genes before applying more computationally expensive methods.
2. Rationale: Filter methods evaluate each feature independently of the classifier, making them computationally efficient and suitable for very high‑dimensional data [17,18].
3. Procedure:
  - For each one‑vs‑rest task, the importance of each gene was assessed using a univariate statistical test.
  - An ANOVA F‑test [18] was applied to compare the mean expression values between the target class and the remaining samples.
  - Genes with low statistical significance or low discriminative power were removed from the feature set.
4. Outcome: A reduced set of genes passed to the next phase. This step ensured only potentially relevant features entered the more demanding supervised selection process.

#### Step 2: Filter-based feature selection based on neighbors.

1. Objective: Identify an optimal subset of features that maximizes class‑separability for each one‑vs‑rest classification task.
2. Rationale: Unlike filter methods, supervised methods consider relationships between features and their joint effect on classification, improving the relevance of the final feature subset.
3. Procedure:
  - The Relief algorithm [19] was applied to the feature subset obtained from the filter phase.
  - Relief evaluates each gene by comparing near‑neighbor samples from the same and opposing classes, assigning higher scores to features that best distinguish the target class from others.
  - Features were ranked according to the Relief score, and the top‑ranked genes were retained as the final selected features.
4. Outcome: A compact, discriminative subset of genes for each binary classification task (High vs. Rest, Medium vs. Rest, Low vs. Rest). These selected genes were subsequently used for graph construction and model training.

The feature selection pipeline was implemented using the following Python libraries: OS, pickle, pandas, scikit‑learn, matplotlib, seaborn, scikit‑feature, and NumPy.

### Fourth step: Classification

**Fig 4:**
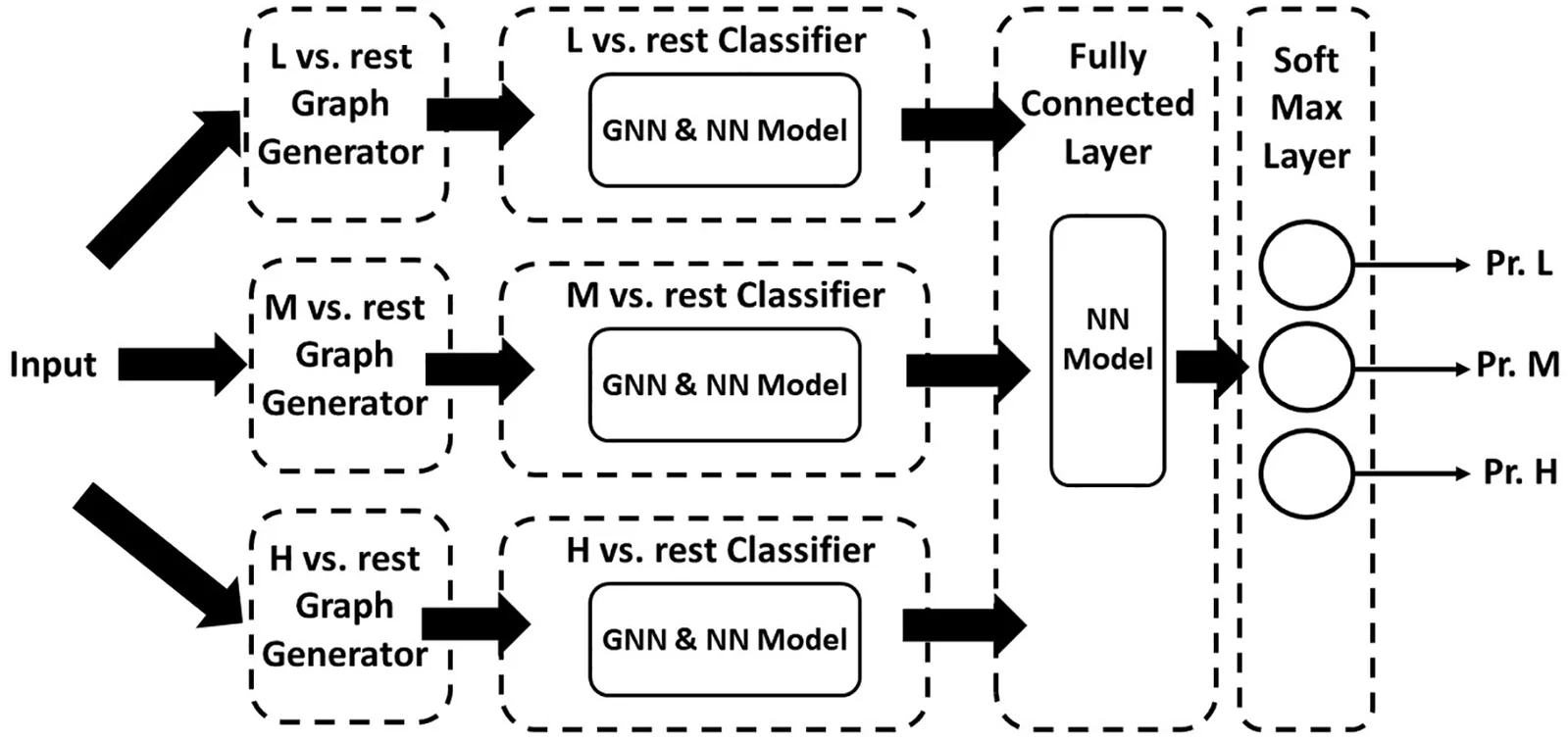
The proposed topology of the final model for classification.

In the fourth step, a classification framework was developed to evaluate whether the genes selected in the previous step can effectively discriminate among prostate cancer risk groups. The classification task was formulated using a one‑vs‑rest strategy, resulting in three binary classification settings: Low vs. Rest, Medium vs. Rest, and High vs. Rest. An overview of the full workflow for this step is shown in Fig 4.

#### Graph construction.

For each patient sample, a graph representation was constructed based on the selected genes. In these graphs, nodes correspond to the selected genes, while edges represent gene–gene relationships. The connectivity between genes was determined using the GeneMANIA database, where edges were defined according to gene co‑expression relationships. As a result, all samples share the same graph topology, while the node features vary across samples.

**Fig 5:**
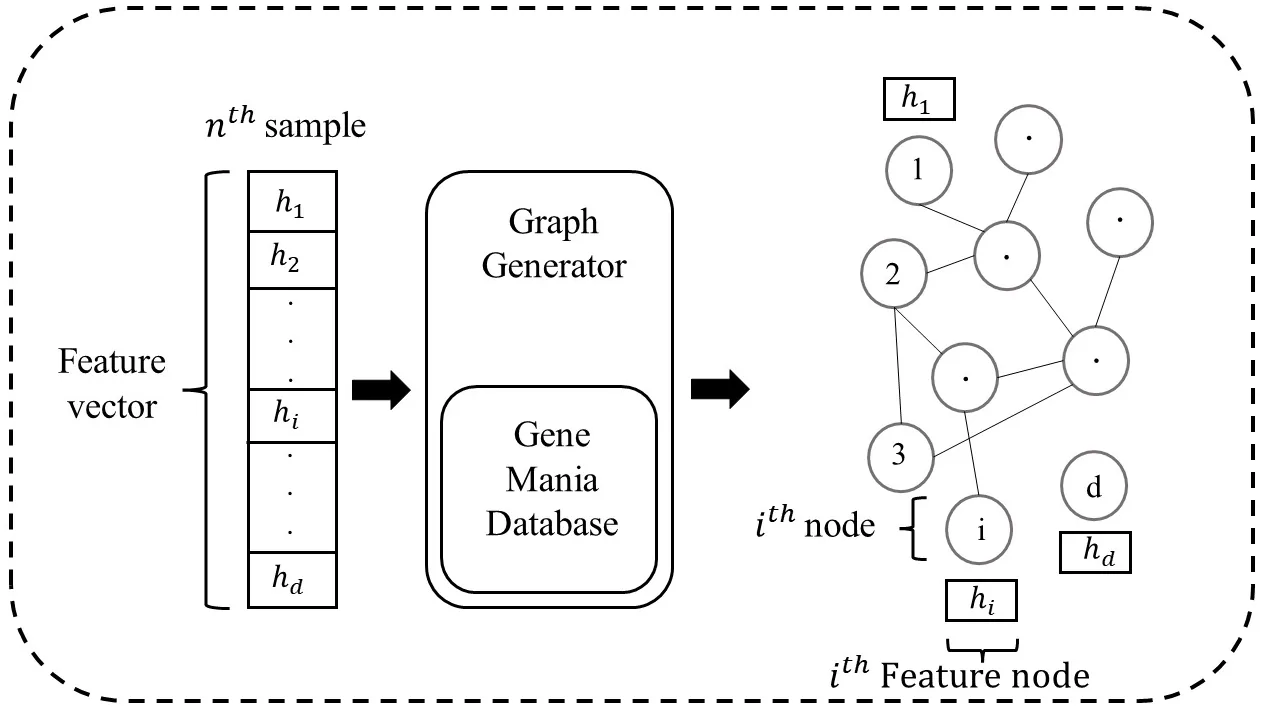
The structure of each graph is related to each sample.

Each node was assigned a feature corresponding to the gene expression value of that gene in the given sample. This representation allows the model to capture both gene expression information and the biological relationships between genes. This process is displayed in Fig 5.

#### GNN & NN model architecture.

The proposed classification model consists of two complementary branches designed to capture different aspects of the data:

1. Graph Neural Network Branch

The first branch processes the graph-structured data using a Graph Attention Network (GAT) [20]. The GAT model learns node representations by assigning attention weights to neighboring nodes, allowing the model to emphasize more informative gene–gene interactions. The GNN branch produces a graph-level embedding vector that summarizes the structural and expression information contained in the gene interaction network.

2. Neural Network Branch

In parallel, the second branch processes the original feature vector of each sample using a standard feed-forward neural network. This branch learns a complementary representation directly from the gene expression data without explicitly considering the graph structure.

3. Feature Fusion and Prediction

**Fig 6:**
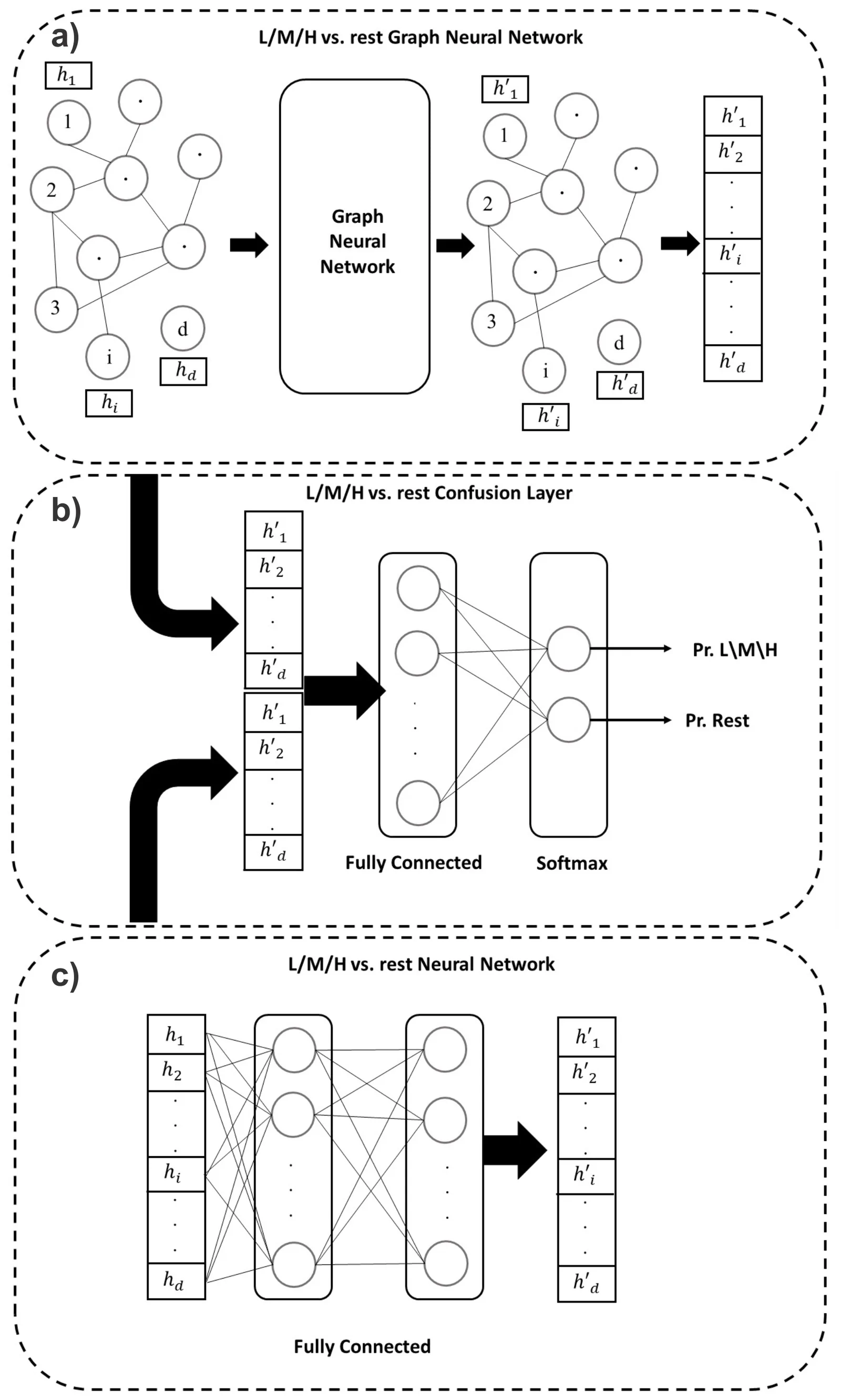
The structure of the GNN & NN sub-model, (a) First part: Graph Neural Network, (b) Third part: Fully Connected fusion layer, (c) Second part: Feedforward Neural Network.

The output representations produced by the GNN and neural network branches are concatenated to form a unified feature vector. This fused representation is then passed through additional fully connected layers to perform classification. A Softmax activation function is applied in the final layer to generate the predicted class probabilities. An overview of the sub-model (one‑vs‑rest) is shown in Fig 6.

#### Training strategy.

Three independent sub-models were trained corresponding to the three one‑vs‑rest classification tasks (Low vs. Rest, Medium vs. Rest, and High vs. Rest). Each sub-model was first pre-trained on its respective dataset. After this stage, the sub-models were integrated into a final classification framework where their outputs were combined and further processed through a fully connected neural network to produce the final prediction.

The classification results obtained from this step were used to assess the discriminative capability of the selected gene features for prostate cancer risk stratification. In this step, the following libraries were utilized: OS, Pickle, Pandas, Scikit-learn, Matplotlib, Seaborn, PyTorch, DGL, and NumPy.

### Fifth step: Interpretation

The purpose of this step is to identify features that play a critical role among the relevant feature set obtained in earlier steps, enabling further investigation in the laboratory by genetics experts. After evaluating the selected feature set in this step, features that significantly contribute to the underlying mechanism of each risk group are extracted using the “SHapley Additive exPlanations” (SHAP) method [21]. The “shap” library in Python was utilized for computing SHAP values and visualizing the interpretable results.

## Results

**Fig 7:**
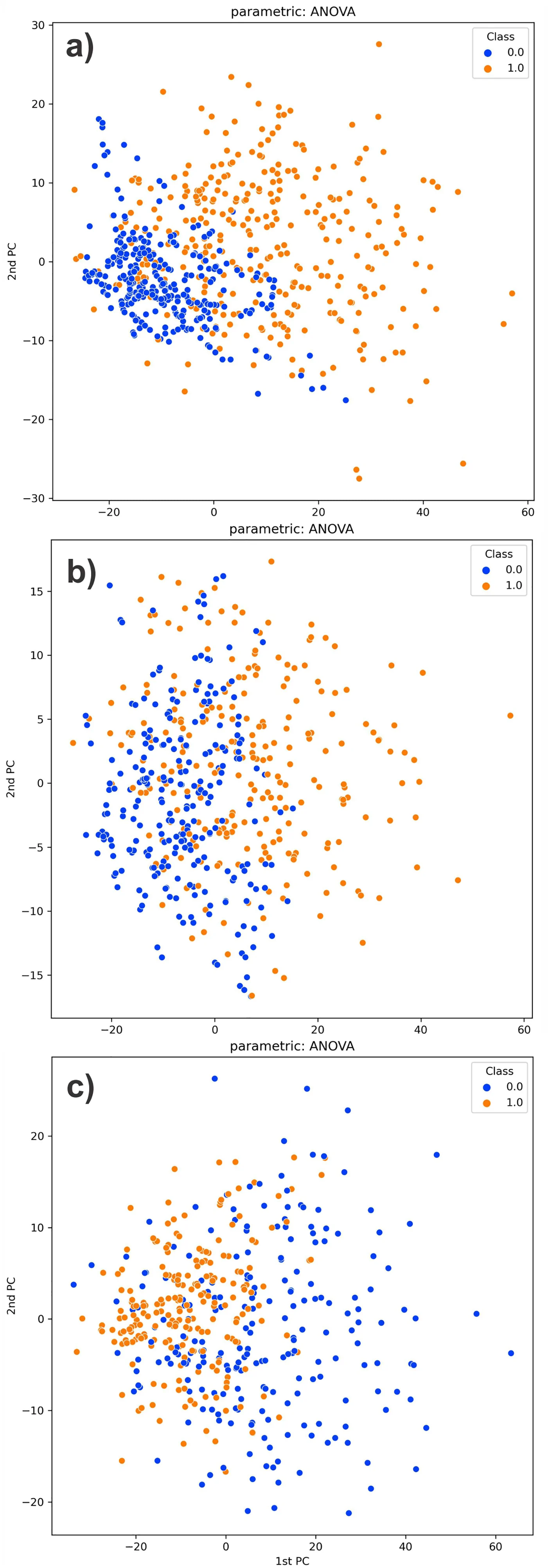
The visualization of the training set based on 1000 selected features (ANOVA method) using the two dimensions of the PCA method, (a) Low (zero class) vs. rest group (1 class), (b) Medium (zero class) vs. rest group (1 class), (c) High (zero class) vs. rest group (1 class). See Supplementary S2 File (section a).

The ANOVA filter method, which is based on parametric statistical tests, was utilized for the initial feature screening. This filter method was then applied to the training data matrix, resulting in the selection of 100, 500, and 1,000 significant features based on the p-values calculated in the statistical tests. To determine the optimal number of features during this phase, visualization method Principal Component Analysis (PCA) along with a Support Vector Machine (SVM) classifier model were employed to examine and assess the separability of risk groups among prostate cancer patients. Fig 7 displays the PCA visualization outputs, and Table 6 presents the classifier model’s prediction based on the training and validation data. Based on the plots and the results from the classifier model, selecting 1000 features with the lowest p-value was recommended in this phase.

**Table 6:** The performance of SVM based on the selected 1000 features (ANOVA method).

Groups	Training Data				Validation Data	
	Main data		PCA Data		Main Data	
	Acc. (%)	AUC	Acc. (%)	AUC	Acc. (%)	AUC
Group 1 (L vs. rest)	91	0.91	81.5	0.815	82.5	0.68
Group 2 (M vs. rest)	78.3	0.783	73.2	0.732	72.5	0.755
Group 3 (H vs. rest)	82.5	0.82	73.4	0.734	82.5	0.817

L: low, M: medium, and H: high risk.

See Supplementary S2 (section a) Files.

**Fig 8:**
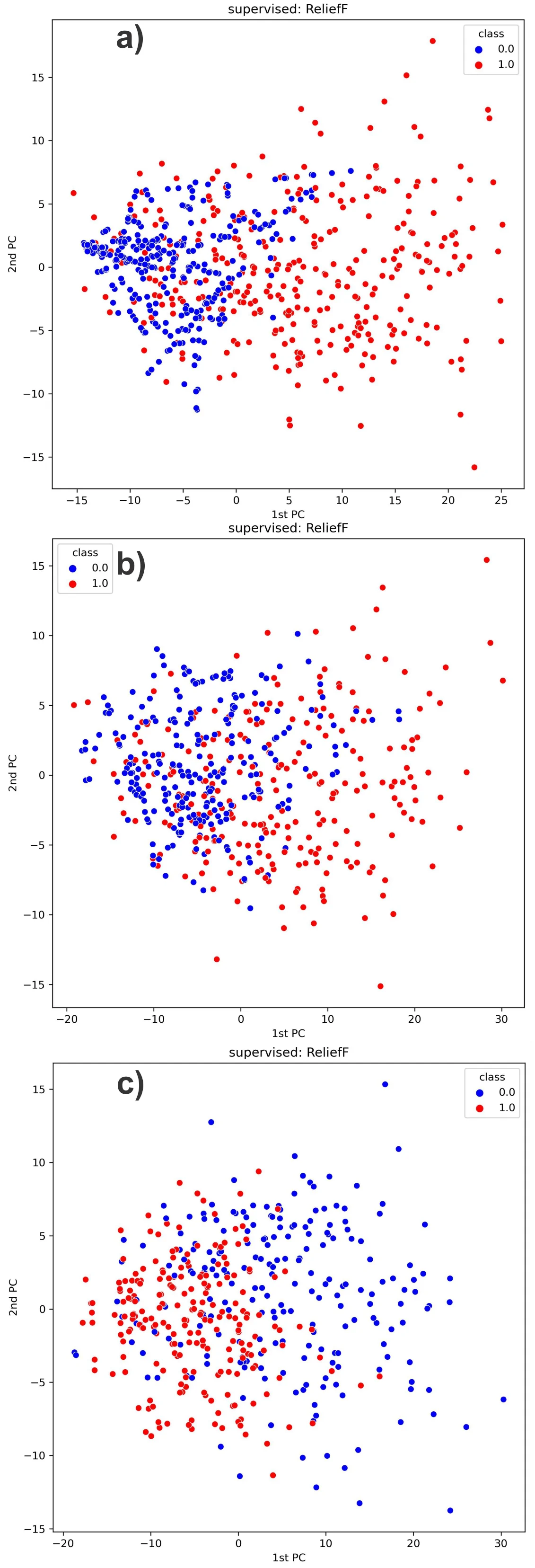
The visualization of the training set based on 100 selected features (ReliefF algorithm) using the two dimensions of the PCA method, (a) Low (zero class) vs. rest group (1 class), (b) Medium (zero class) vs. rest group (1 class), (c) High (zero class) vs. rest group (1 class). See Supplementary S2 File (section b).

Then, the ReliefF algorithm, a supervised method, was used to select the most influential features for each group. The algorithm was applied to the training data matrix, and sets of 50, 100, 150, and 200 top features were selected based on the weights computed by the ReliefF algorithm. Fig 8 presents the PCA visualizations, while Table 7 shows the classification results based on the training and validation set. Based on the visualizations and classification outcomes, it was recommended to select the top 100 features with the highest ReliefF weights.

**Table 7:** The performance of SVM based on the selected 100 features (Relief method).

Groups	Training Data				Validation Data	
	Main data		PCA Data		Main Data	
	Acc. (%)	AUC	Acc. (%)	AUC	Acc. (%)	AUC
Group 1 (L vs. rest)	94.6	0.946	80	0.8	82.5	0.68
Group 2 (M vs. rest)	79.7	0.797	71.8	0.718	70	0.73
Group 3 (H vs. rest)	83.8	0.838	77.2	0.772	82.5	0.811

L: low, M: medium, and H: high risk. See Supplementary S2 (section b) Files.

The specifications of the graph-based input data for the three groups are divided into training, validation, and test sets. Although 100 top features were selected during the feature selection process, the number of graph nodes corresponding to each sample is reported as 91, 90, and 94 for the respective groups. This difference arises because some genes are missing from the Gene-MANIA database, leading to their exclusion from the selected gene set.

The structure of each sub‑model (L/M/H vs. rest) consists of three components. Graph Neural Network (GNN) component: We employed a Graph Attention Network (GAT) with a GAT convolution layer using input dimensions of one feature per node and 91, 90, and 94 nodes for the L vs. rest, M vs. rest, and H vs. rest tasks, respectively. The layer produced a 32‑dimensional output with one attention head. ReLU activation, a dropout layer, and a max‑pooling layer were also incorporated in this component. Neural Network component: This stage used a feed‑forward neural network with 64 input units and 2 output units. Fully connected classification component: Finally, a fully connected layer with 64 input units and 2 output units, followed by a softmax activation function, was applied for classification. Finally, the outputs of the three sub-models were concatenated and fed into a simple feed-forward neural network with 6 input units and 2 output units, followed by a softmax activation function for final classification.

**Fig 9:**
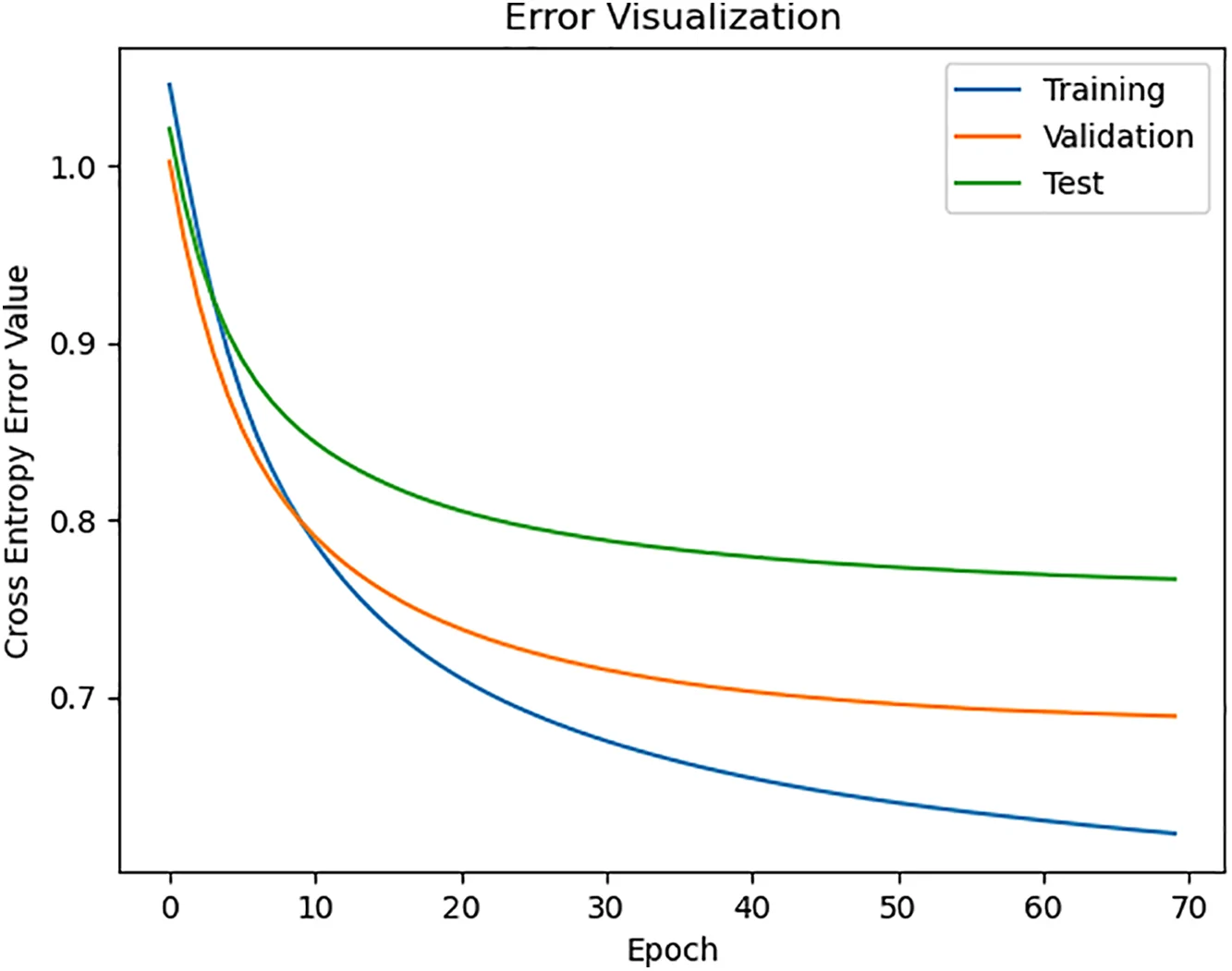
The error plot for training, validation, and test sets. See Supplementary S3 File (section a).

**Fig 10:**
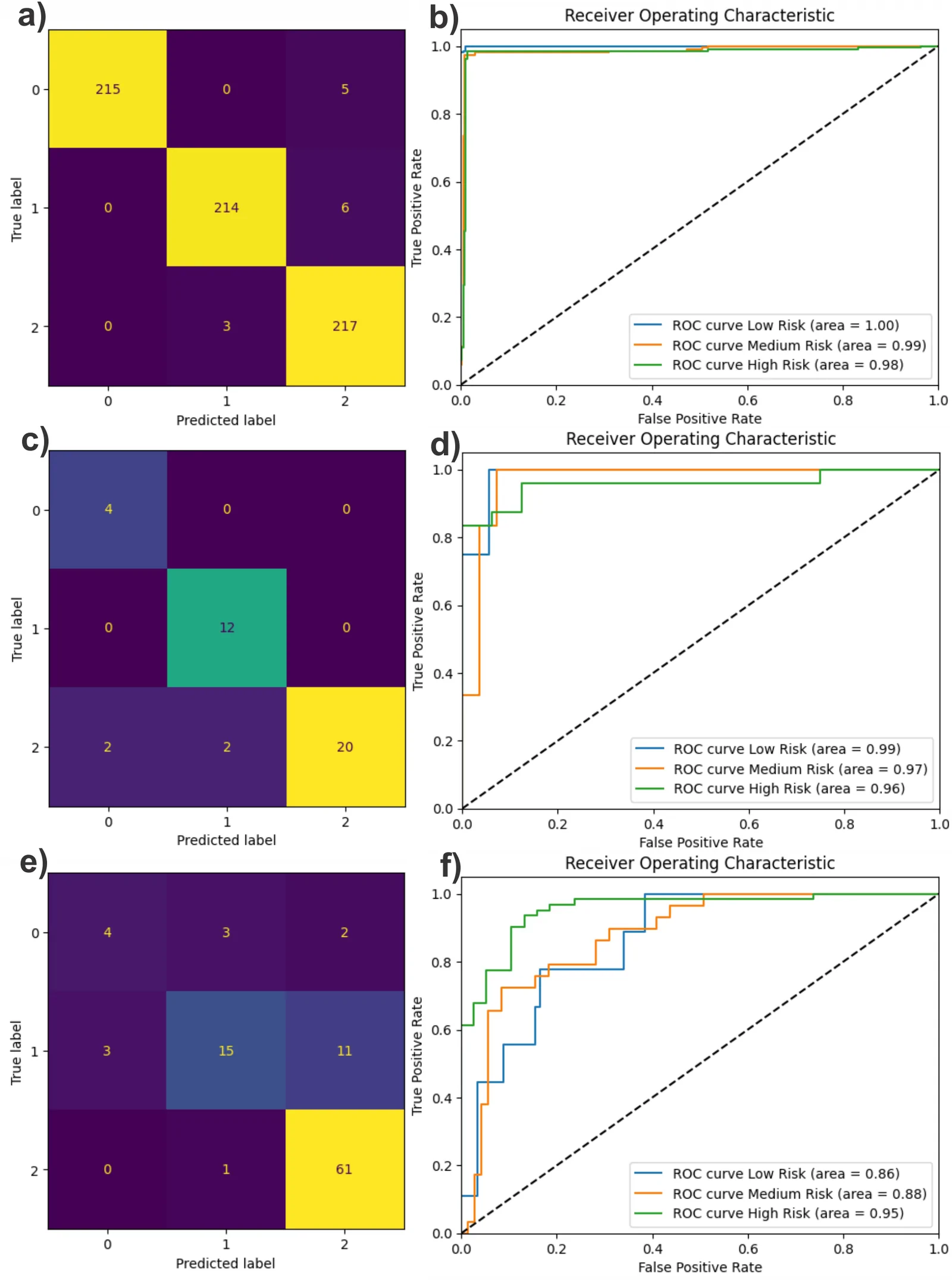
The confusion matrix and ROC plot for (a, b) training set, (c, d) validation set, (e, f) test set. See Supplementary S3 File (section a).

After training the sub-models with the previously discussed graph-based data, these sub-models (Fig 5) were integrated into the final model (Fig 4), followed by the final training process. For training all models, the Cross-Entropy loss function and the Stochastic Gradient Descent (SGD) optimization method with a learning rate of 0.01 were utilized. The results of the final model on the training, validation, and test sets are presented in Table 8. Additionally, the error rates, AUC-ROC curves, and confusion matrices are displayed in Figs 9 and 10, respectively.

**Table 8:** The performance of the proposed classifier model based on the selected features.

	Training	Validation	Test
ACC (%)	99	90	**80**
AUC (L vs. rest)	1	0.99	**0.86**
AUC (M vs. rest)	0.99	0.97	**0.88**
AUC (H vs. rest)	0.98	0.95	**0.95**
Recall (L)	1	1	0.44
Recall (M)	0.97	1	0.52
Recall (H)	0.99	0.83	0.98
Precision (L)	1	0.67	0.57
Precision (M)	0.99	0.86	0.79
Precision (H)	0.97	1	0.82
F1-Score (L)	1	0.8	0.5
F1-Score (M)	0.98	0.92	0.62
F1-Score (H)	0.98	0.91	0.9
AUPRC (L)	0.99	0.91	0.43
AUPRC (M)	0.96	0.91	0.67
AUPRC (H)	0.96	0.97	0.97

L: low, M: medium, and H: high risk.

See Supplementary S3 (section a) Files.

We also applied several well-established classifiers, including Naive Bayes, Linear Discriminant Analysis, Random Forest, K-Nearest Neighbors, and Support Vector Machine, to distinguish low-risk from the rest, medium-risk from the rest, and high-risk from the rest. Finally, after the training process, the performance of each model is reported in Table 9. The parameters of each model were tuned to achieve optimal performance. The selected settings were as follows: SVM used an RBF kernel with C = 0.1 for low-risk vs. rest, C = 0.5 for medium-risk vs. rest, and C = 0.5 for high-risk vs. rest. KNN used k = 5 neighbors. Random Forest used 3 estimators for low-risk vs. rest, and 5 estimators for both medium-risk vs. rest and high-risk vs. rest. In addition, the low-risk vs. rest, medium-risk vs. rest, and high-risk vs. rest datasets were treated separately because feature selection was performed independently for each dataset using the proposed method before expected classification process. The KNN model achieved the best performance in the low-risk vs. rest and medium-risk vs. rest groups, while the SVM model achieved the best performance in the high-risk vs. rest group, based on the AUC metric (Table 9).

**Table 9:** The test set performance of classifier for each type of models based on the selected features.

	Naïve Bayse		LDA		RF		KNN		SVM	
ACC (L vs rest) %	84		84		89		78		86	
ACC (M vs rest) %	78		76		75		77		78	
ACC (H vs rest) %	82		83		78		83		86	
AUC (L vs. rest)	0.66		0.66		0.73		**0.78**		0.72	
AUC (M vs. rest)	0.75		0.72		0.72		**0.79**		0.74	
AUC (H vs. rest)	0.8		0.8		0.75		0.83		**0.84**	
	L/M/H	Rest	L/M/H	Rest	L/M/H	Rest	L/M/H	Rest	L/M/H	Rest
Recall (L vs. rest)	0.44	0.88	0.44	0.88	0.56	0.92	0.78	0.78	0.56	0.89
Recall (M vs. rest)	0.69	0.82	0.62	0.82	0.66	0.79	0.86	0.73	0.66	0.83
Recall (H vs. rest)	0.89	0.71	0.9	0.71	0.85	0.66	0.81	0.87	0.92	0.76
Precision (L vs. rest)	0.27	0.94	0.27	0.94	0.42	0.95	0.29	0.97	0.33	0.95
Precision (M vs. rest)	0.61	0.87	0.58	0.84	0.56	0.85	0.57	0.93	0.61	0.86
Precision (H vs. rest)	0.83	0.79	0.84	0.82	0.8	0.74	0.91	0.73	0.86	0.85
F1-Score (L vs. rest)	0.33	0.91	0.33	0.91	0.48	0.94	0.39	0.87	0.42	0.92
F1-Score (M vs. rest)	0.65	0.84	0.6	0.83	0.6	0.82	0.68	0.82	0.63	0.84
F1-Score (H vs. rest)	0.86	0.75	0.87	0.76	0.83	0.69	0.85	0.8	0.89	0.81

LDA: Linear Discriminant Analysis, RF: Random Forest, KNN: K-Nearest Neighbors, SVM: Support Vector Machine.

L: low, M: medium, and H: high risk. See Supplementary S3 (section b) Files.

The extraction of highly influential genes from the previously selected features was conducted based on SHAP values. The results, including the names of key contributing genes, were presented along with suitable visualizations. This method was applied to the selected genes for the three groups, and the findings were reported separately.

### Low vs. Rest group

**Fig 11:**
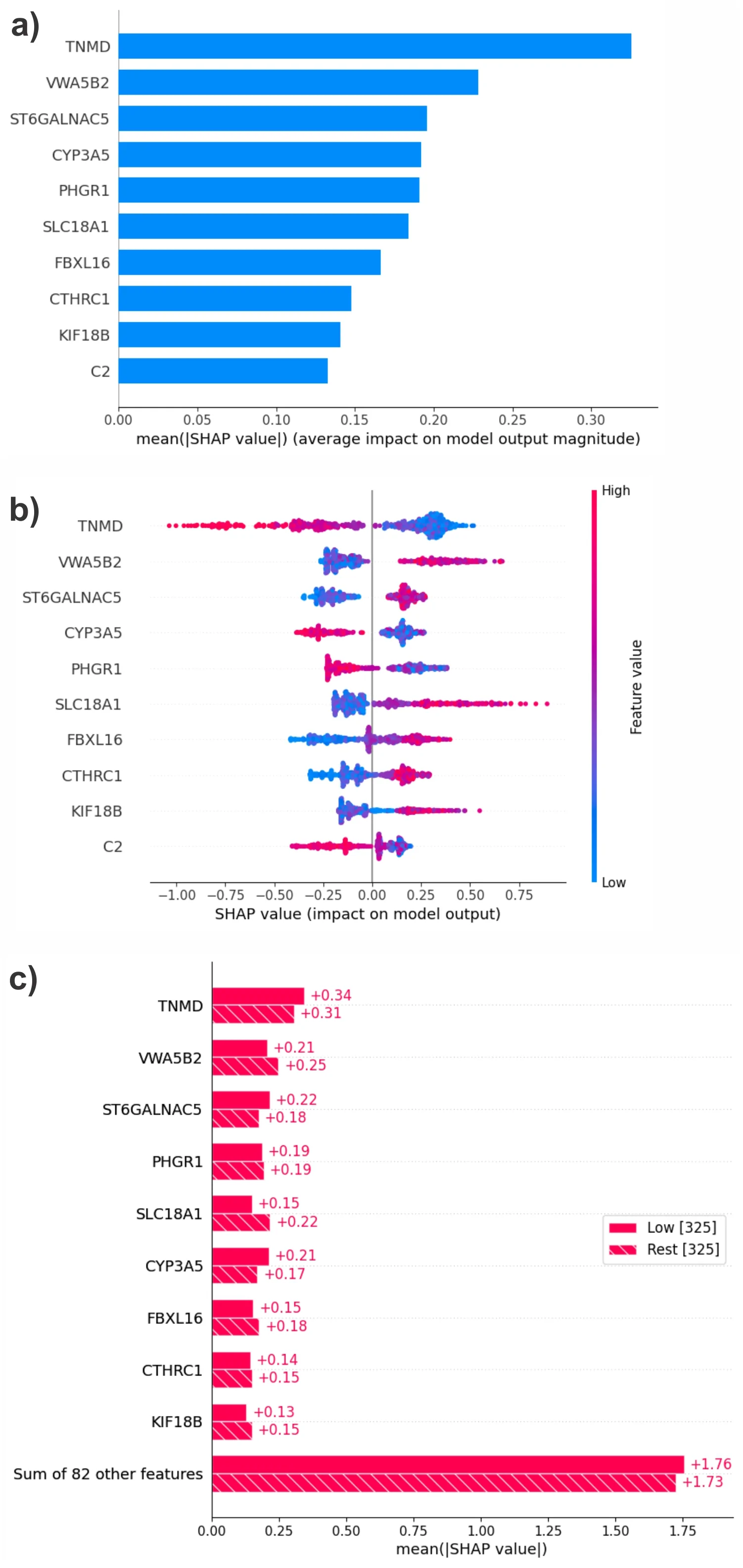
Interpretability and feature importance analysis of the XGBoost model using SHAP values. **(a) Global Feature Importance Bar Plot:** Ranking of the top 10 genes based on their mean absolute SHAP values (mean (∣SHAP∣). This metric represents the average magnitude of each gene’s contribution to the model’s prediction. TNMD emerged as the most influential feature, followed by VWA5B2 and ST6GALNAC5. **(b) SHAP Summary (Bee swarm) Plot:** Distribution of SHAP values for the top 10 genes, illustrating the relationship between gene expression levels and model output. Each point represents an individual sample. The color gradient indicates the feature value (red = high expression, blue = low expression), while the horizontal position indicates the SHAP value. For instance, high expression of TNMD (red) correlates with a negative impact on the model output, whereas high expression of VWA5B2 correlates with a positive impact. **(c) Cohort-Stratified Feature Importance:** Comparison of the mean absolute SHAP values between the “Low” (n = 325) and “Rest” (n = 325) cohorts. The consistent magnitude of importance across both groups suggests that the model’s reliance on these key genes is stable across different sample subpopulations. The bottom bar accounts for the cumulative impact of the remaining 82 features, highlighting that while individual contributions decrease, the collective influence of the genome-wide signature remains significant. See Supplementary S4 File.

SHAP is a method to explain individual predictions of machine learning models by calculating the contribution (or impact) of each feature. The mean (|SHAP value|) measures how much, on average, a feature contributes to the model’s prediction across all samples. To gain a granular understanding of the molecular drivers within our XGBoost model, we performed SHAP-based interpretability analysis (Fig 11). Global feature ranking identified TNMD as the most critical predictor, possessing the highest mean absolute SHAP value (Fig 11a). Analysis of the SHAP summary plot (Fig 11b) revealed the directionality of these effects; for example, TNMD and CYP3A5 showed an inverse relationship with the model’s predicted probability, whereas VWA5B2, ST6GALNAC5, and SLC18A1 demonstrated a positive correlation between high expression and model output.

**Fig 12:**
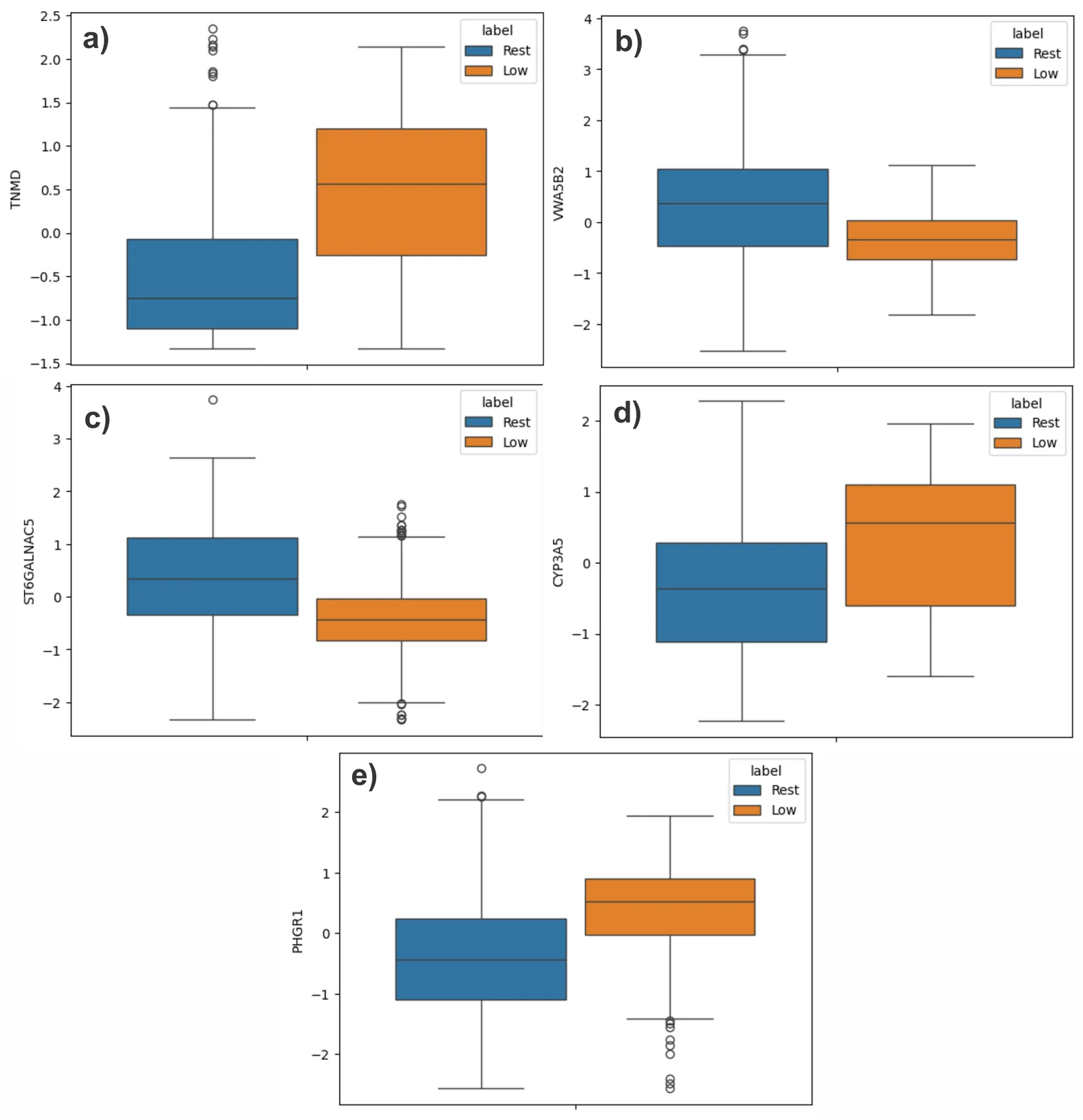
The box plot of the five top genes based on the expression value in Low-risk vs rest group: (a) TNMD, (b) VWA5B2, (c) ST6GALNAC5, (d) CYP3A5, (e) PHGR1.

To ensure the robustness of these findings, we stratified the feature importance by cohort group (Fig 11c). The relative importance of the top genes remained remarkably consistent between the “Low” and “Rest” cohorts, indicating that the model’s decision-making process is not biased toward a specific subgroup but rather relies on a stable genetic signature. While the top 10 genes provided the most significant individual contributions, a substantial portion of the predictive power was derived from the aggregate effect of the remaining 82 features, emphasizing the polygenic nature of the underlying biological condition. Finally, the box plot of the five top genes based on their expression values is displayed in Fig 12.

### Medium vs. Rest group

**Fig 13:**
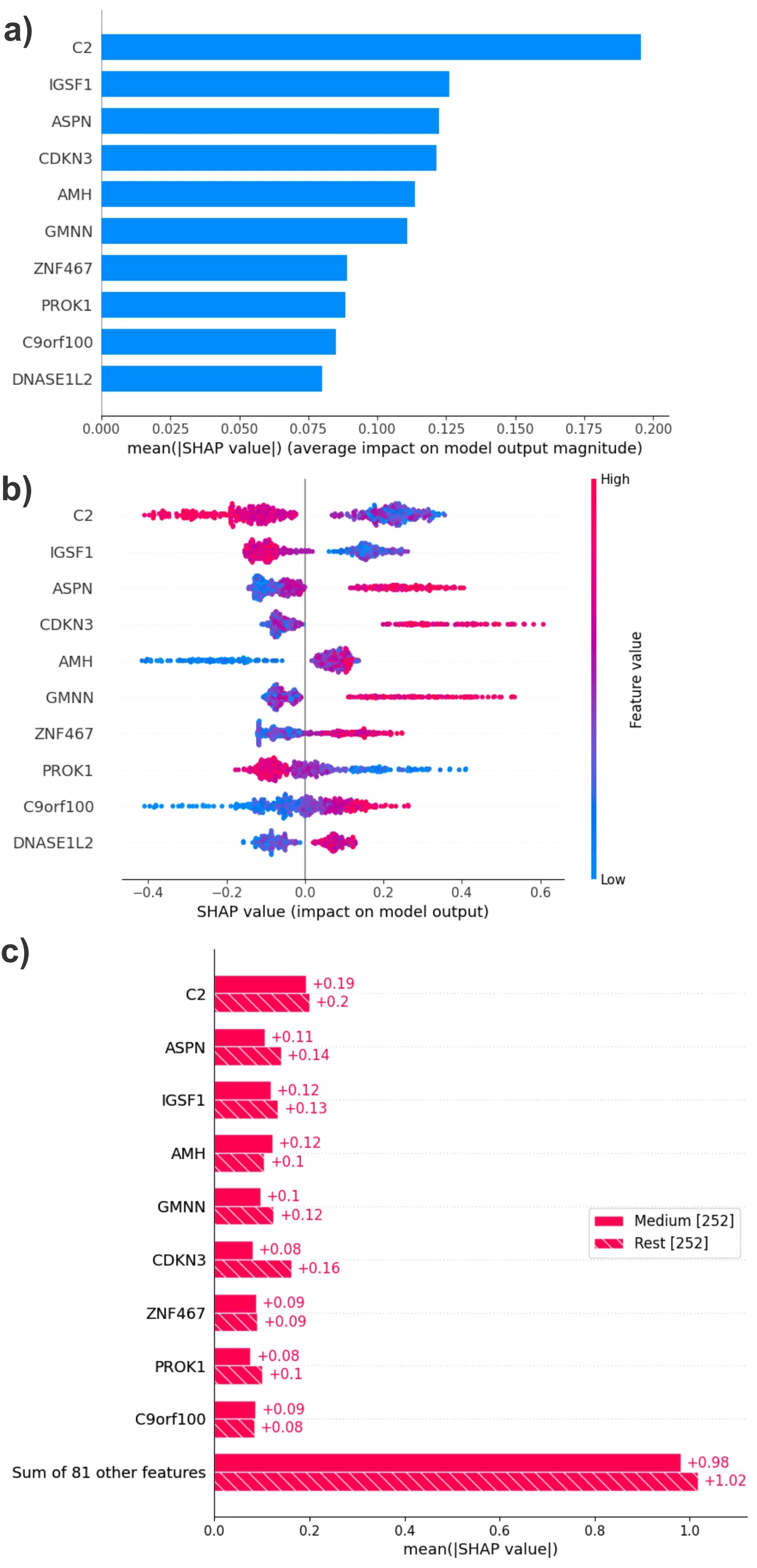
Comprehensive SHAP-based interpretability of the XGBoost model for the Medium cohort. **(a) Global Feature Importance:** Ranking of genes based on the mean absolute SHAP value (mean(∣SHAP∣). This plot quantifies the average contribution of each feature to the final classification. C2 was identified as the most influential feature, followed by IGSF1 and ASPN. **(b) SHAP Summary (Bee swarm) Plot:** Visualization of the top 10 genes ranked by their impact on model predictions. The horizontal axis represents the SHAP value (impact on output), while the color gradient indicates gene expression levels (red = high, blue = low). Genes such as ASPN, CDKN3, and GMNN show a positive correlation with model output (high expression increases the prediction), while C2 and IGSF1 demonstrate an inverse relationship. **(c) Cohort-Specific Feature Importance (Medium vs. Rest):** Comparison of feature importance magnitudes between the “Medium” group (n = 252) and the remaining samples (“Rest”, n = 252). The high degree of symmetry between the two groups indicates that the model utilizes a stable and consistent set of features across different data subsets, further validating the robustness of the identified genetic markers. See Supplementary S4 File.

To further explore the stability of our predictive model within specific clinical or biological subgroups, we performed a SHAP interpretability analysis on the “Medium” cohort (Fig 13). The global feature importance ranking highlighted C2 as the most significant predictor (mean (∣SHAP∣ ≈ 0.19), followed by IGSF1 and ASPN (Fig 13a).

The SHAP summary bee swarm plot (Fig 13b) provided insight into the directionality of these effects. Specifically, we observed that high expression levels of ASPN, CDKN3, AMH, and GMNN were associated with positive SHAP values, contributing to an increased model output. Conversely, C2 and IGSF1 exhibited a negative correlation, where lower expression levels tended to drive higher predictive scores.

**Fig 14:**
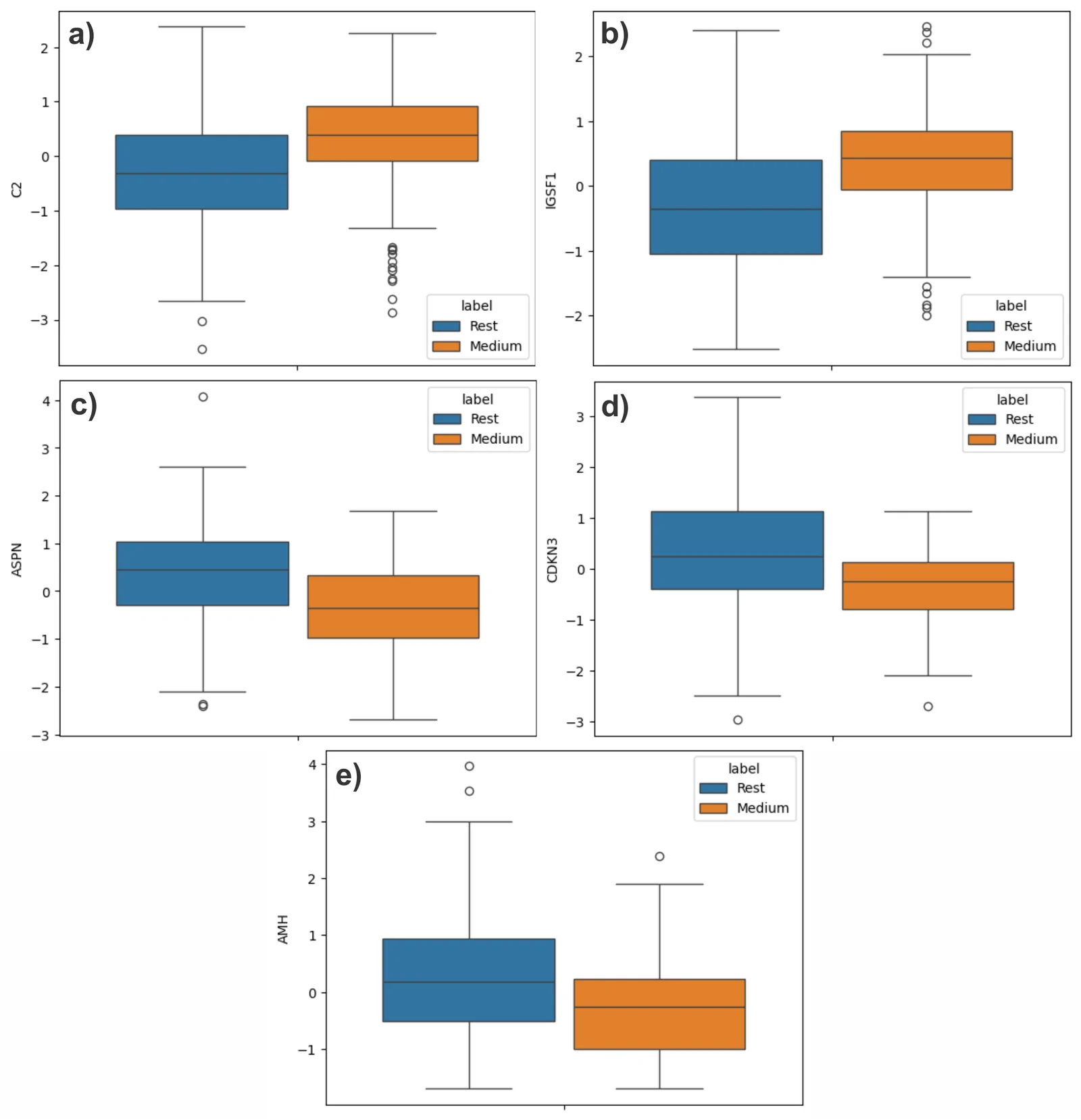
The box plot of the five top genes based on the expression value in Medium-risk vs rest group: (a) C2, (b) IGSF1, (c) ASPN, (d) CDKN3, (e) AMH. See Supplementary S4 File.

Finally, to assess the generalizability of these findings, we compared the feature importance distributions between the “Medium” and “Rest” groups (Fig 13c). The nearly identical importance magnitudes for the top-ranking genes across both cohorts demonstrate that the model’s decision-making process is consistent and not driven by outliers or group-specific biases. Notably, while the top 10 genes provided the most interpretable insights, the cumulative effect of the remaining 81 features contributed significantly to the model’s overall performance, reflecting the complex, polygenic architecture of the studied condition. Finally, the box plot of the five top genes based on their expression values is displayed in Fig 14.

### High vs. Rest group

**Fig 15:**
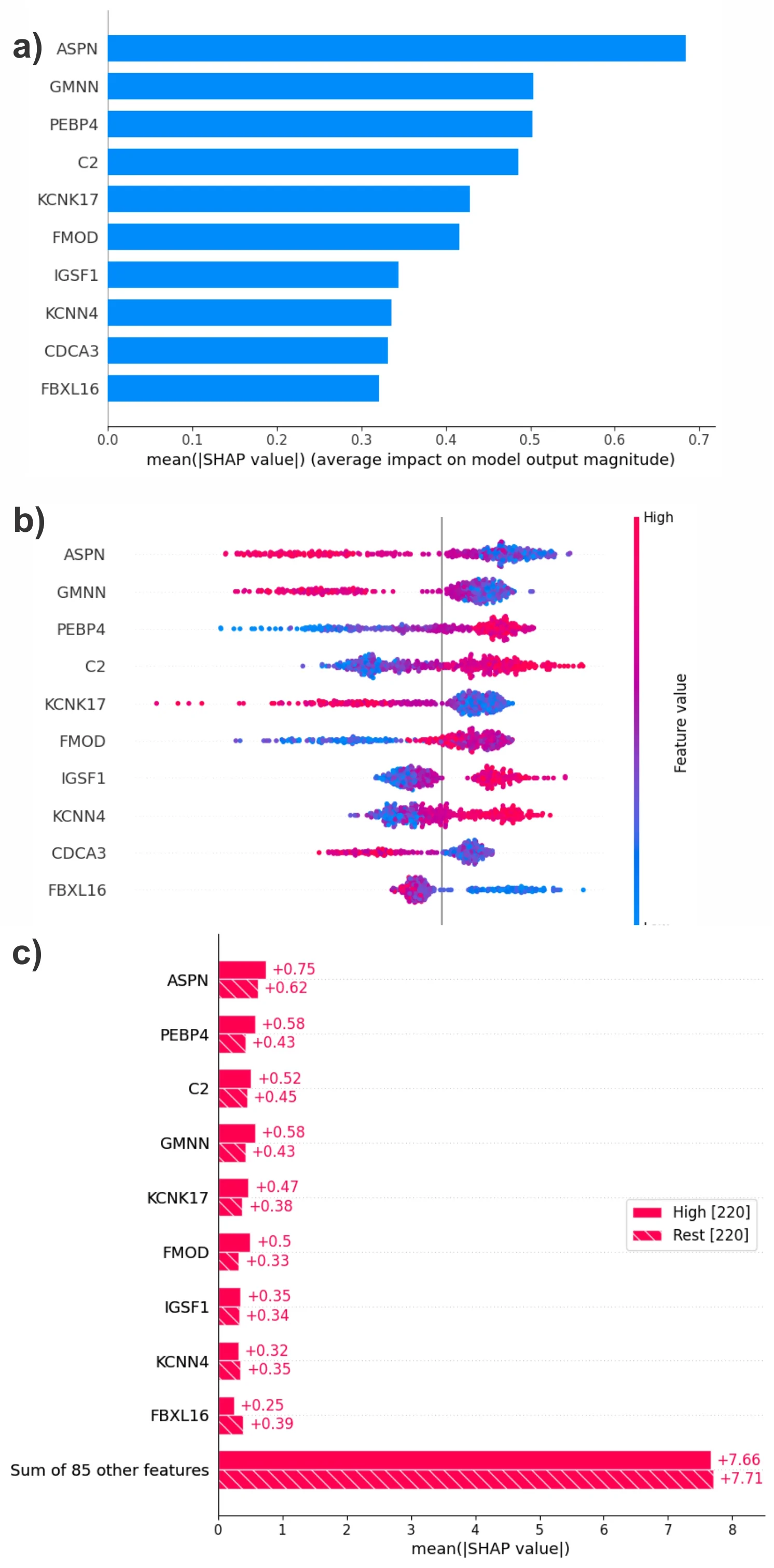
SHAP-based model interpretability for the High cohort. **(a) Global Feature Importance Bar Plot:** Displays the ranking of the top 10 genes based on their mean absolute SHAP values (mean (∣SHAP∣)), which quantifies the average magnitude of their contribution to the model’s prediction. ASPN is identified as the most impactful feature, followed by GMNN and PEBP4. See Supplementary S4 File. **(b) SHAP Summary (Bee swarm) Plot:** Depicts the top 10 most influential genes as determined by their SHAP values, illustrating their impact on the model’s output. Each point corresponds to an individual sample, with its color reflecting the gene expression level (red = high, blue = low). For instance, high expression of ASPN and GMNN generally drives positive model predictions, while high expression of PEBP4 and KCNK17 tends to exert a negative influence. **(c) Cohort-Stratified Feature Importance (High vs. Rest):** Comparison of the mean absolute SHAP values between the “High” cohort (n = 220) and the “Rest” cohort (n = 220). The consistent feature importance observed across both groups highlights the robustness of the identified genetic signature, indicating that the model’s predictive mechanism is stable irrespective of cohort stratification. The cumulative SHAP value of the remaining 85 features underscores the polygenic nature of the underlying biological process. See Supplementary S4 File.

To dissect the driving molecular features within the “High” cohort, we performed an in-depth SHAP interpretability analysis of our XGBoost model (Fig 15). The global feature importance analysis (Fig 15a) identified ASPN as the most critical gene, demonstrating the highest mean absolute SHAP value, followed by GMNN and PEBP4.

Further examination of the SHAP summary (bee swarm) plot (Fig 15b) revealed the directional impact of these genes. High expression levels of ASPN, GMNN, C2, FMOD, IGSF1, KCNN4, and CDCA3 were consistently associated with positive SHAP values, contributing to an increased model output. Conversely, low expression of PEBP4 and high expression KCNK17 typically exerted a negative influence on the model’s prediction.

**Fig 16:**
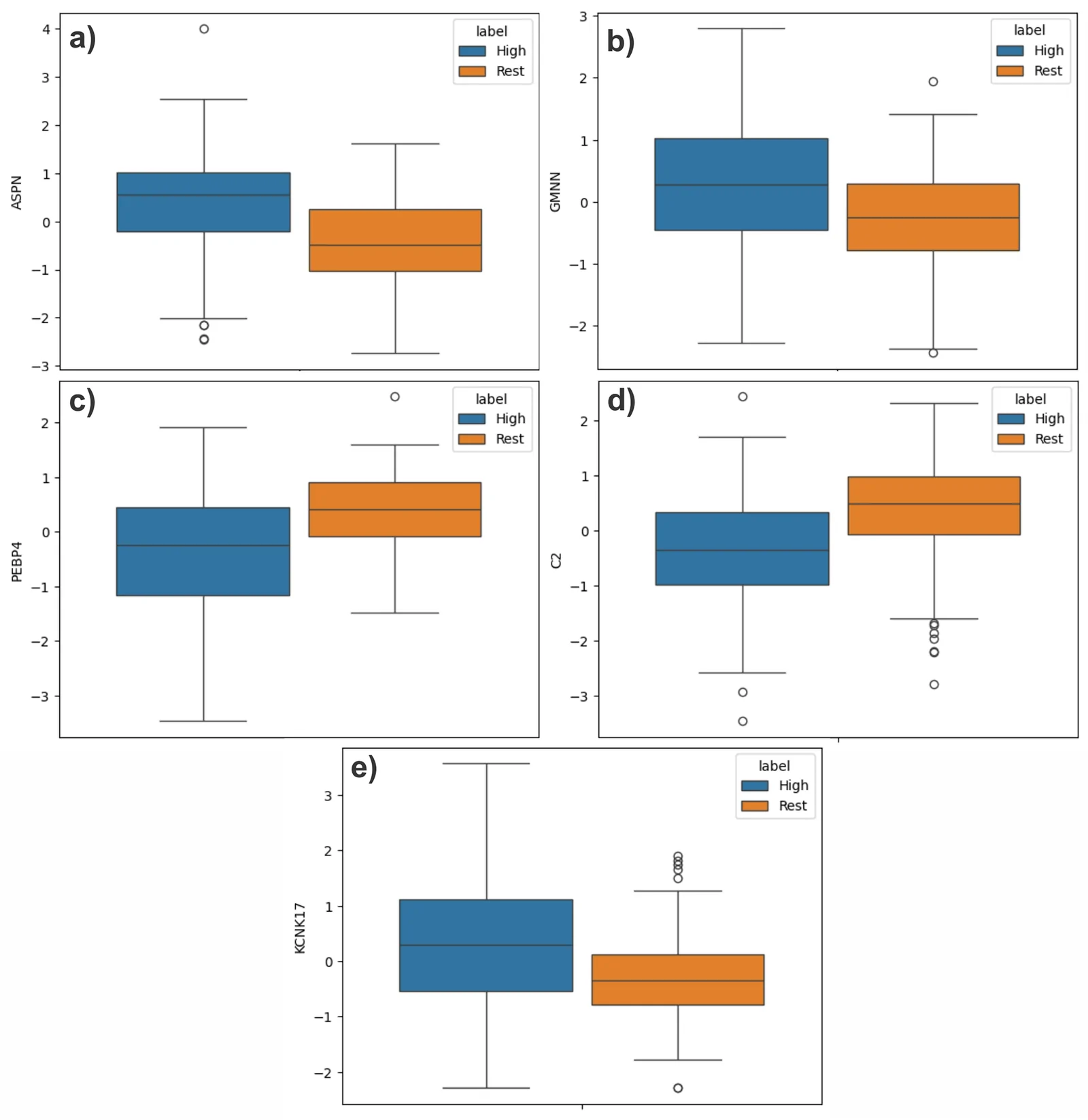
The box plot of the five top genes based on the expression value in High-risk vs rest group: (a) ASPN, (b) GMNN, (c) PEBP4, (d) C2, (e) KNCK17. See Supplementary S4 File.

To validate the generalizability and stability of these findings, we compared the feature importance between the “High” cohort and the “Rest” cohort (Fig 15C). The striking similarity in the mean absolute SHAP values across both groups confirms that the model’s reliance on these key genes is consistent and not specific to a particular subgroup. This stability supports the robustness of the identified genetic markers. While individual top features showed substantial influence, the considerable aggregate SHAP value from the remaining 85 features (+7.66 for High, + 7.71 for Rest) suggests a complex, distributed predictive network across the transcriptome. Finally, the box plot of the five top genes based on their expression values is displayed in Fig 16.

## Discussion

Combining GNN-based modeling with AI analysis revealed distinct mRNA expression profiles associated with prostate cancer aggressiveness, as grouped by Gleason Score. Differentially expressed genes were identified in the High (ASPN, GMNN, PEBP4, C2, KNCK17), Medium (C2, IGSF1, ASPN, CDKN3, AMH) and Low (TNMD, VWA5B2, ST6GALNAC5, CYP3A5, PHGR1) groups, emphasizing the molecular heterogeneity in the progression of disease.

Asporin (ASPN) is a small secretory extracellular matrix proteoglycan that is enriched in leucine. It is involved in regulating signaling pathways crucial for tissue homeostasis and cancer progression [22,23]. In prostate cancer, Asporin is mainly expressed by cancer-associated fibroblasts (CAFs) in reactive stroma (a histopathological feature of aggressive subtypes), promoting tumor progression, invasion, and metastasis. Its unique structure and D-repeat polymorphisms can impact its function and interactions as an inherited modulator of metastatic prostate cancer [24]. TGFβ activates ASPN and ASPN enhances β-catenin expression and facilitates its nuclear accumulation through activation of the Wnt/β-catenin signaling pathway, promoting stemness and epithelial–mesenchymal transition (EMT) in prostate cancer. Asporin may also upregulate matrix metalloproteinases to aid tissue invasion by enhancing FGF2 activity. As an upstream regulatory factor, ASPN also enhances docetaxel resistance and metastasis by activating Wnt/β-catenin signaling [25]. Moreover, elevated mRNA and protein levels of ASPN [25–28] in patients with prostate cancer are associated with shorter time to biochemical recurrence after radical prostatectomy, making it an independent prognostic marker alongside Gleason score and clinical stage. Patients with a Gleason score greater than 7 and high ASPN expression had a 41% biochemical recurrence rate, compared to 22% in those with lower expression [26]. In the present study, the identification of Asporin as a top dysregulated gene in both the High and Medium prostate cancer groups underscores its key involvement in tumor aggressiveness and progression, influencing stromal-tumor interactions, extracellular matrix remodeling, and metastatic potential. The absence of significant dysregulation in the Low group also suggests that ASPN upregulation may be specifically associated with the transition to more aggressive phenotypes, aligning with clinical observations that link higher ASPN levels to poorer outcomes and biochemical recurrence. These findings support targeting ASPN-related pathways as a promising avenue for therapeutic intervention aimed at halting or reversing the progression of clinically significant prostate cancer.

Geminin (GMNN) is encoded by the *GMNN* gene, located on chromosome 6. By binding and inhibiting CDT1, GMNN restricts replication of DNA to once per cell division. This action mainly occurs in the S and G2 phases of the cell cycle. Geminin also modulates the activity of Topoisomerase IIα (TOP2A) during chromosome segregation. These regulations are critical for maintaining genome stability and proper cell cycle progression. Over-expression of GMNN can induce genomic instability and resistance to TOP2A-targeting chemotherapies [29]. It is reported that SPOP (Speckle-type POZ protein) controls Geminin poly-ubiquitination and prevents aberrant DNA re-replication in normal cells. Dysfunctional Geminin regulation (especially via SPOP mutations, that is common in prostate cancer) increases replication errors and genome instability in prostate cancer, promoting aggressive disease [30]. In cancerous prostate cells, the expression level of GMNN is elevated compared to normal tissues. High GMNN expression is associated with tumor aggressiveness, metastasis, and poorer outcomes and can predict recurrence and metastasis in conjunction with the Gleason Score [31–33]. Depletion of GMNN in cancer cells triggers DNA damage and spontaneous apoptosis, arresting cancer cell proliferation without affecting normal cells. This selective vulnerability happens because many cancer cells rely solely on geminin to suppress CDT1 activity and prevent DNA re-replication. In contrast, normal cells have additional safeguards to prevent this lethal re-replication, highlighting the cancer-specific dependency on geminin [34]. Likewise, targeting CDT1/Geminin complex [35] represents a possible strategy for selective cancer therapy. Hence, targeting GMNN could be a strategy to inhibit tumor growth and metastasis in prostate cancer.

PEBP4 (phosphatidylethanolamine-binding protein 4) is an anti-apoptotic protein that regulates cancer cell proliferation and invasion mainly through modulating pro-apoptotic and PI3K/Akt signaling pathways [36]. PEBP4 is upregulated under hypoxic conditions in prostate cancer, promoting epithelial-to-mesenchymal transition (EMT), a critical step in cancer metastasis. Targeting PEBP4 has been proposed as a therapeutic strategy, especially for resistant tumors to standard therapies such as tumor necrosis factor (TNF-α) and TNF-related apoptosis-inducing ligand (TRAIL). Silencing PEBP4 inhibits hypoxia-induced EMT, reduces cell migration and invasion, suggesting that it is a key mediator of prostate cancer aggressiveness under hypoxic tumor microenvironments [37]. Moreover, a small molecule inhibitor, by binding to and inhibiting hPEBP4, restores apoptosis in resistant prostate cancer cells [38]. Collectively, PEBP4 is involved in prostate cancer progression and may serve as both a diagnostic marker (due to its elevated expression in cancer tissues and cell lines) and a potential therapeutic target (through its influence on EMT and oncogenic signaling pathways) [37,38].

The GNN-based framework identified C2 as a shared marker between the High and Medium-risk groups, suggesting a potential biological continuity between these stages. C2 is an inflammation protein involved in the classical complement pathway of innate immunity. In Melanoma, tumoral C2 by stimulating an anti-tumor M1 macrophage subtype over M2 (pro-tumor M2 macrophage subtype) controls the tumor microenvironment [39]. While direct clinical studies on C2 alone in prostate cancer are scarce, its role in complement activation and the associated immune and inflammatory modulation are important to understand how innate immunity impacts prostate cancer biology and therapy resistance.

KNCK17 gene (also known as KIN17) encodes a protein involved in DNA and RNA binding that is involved in DNA repair and cellular processes relevant to cancer broadly [40]. Its direct connection to prostate cancer is not established in current studies.

IGSF1 (immunoglobulin superfamily member 1) was identified as the second key gene in the Medium-risk group. It is an immune suppressor primarily expressed in tumors, but not in normal tissues [41]. Increased levels of IGSF1 have also been reported in biopsy samples of patients with prostate cancer [42,43]. Its inclusion in a molecular panel could enhance early diagnosis and assessment of prostate cancer stage. As an immune target, IGSF1 may also complement present cancer immunotherapeutics [41]. However, more research is needed to clarify its biological effects on prostate cancer.

CDKN3 (cyclin-dependent kinase inhibitor 3) is a cell cycle regulator that regulates cell division by dephosphorylating CDK2 and CDK1. CDKN3 exerts broad tumor-promoting effects that may be linked to mechanisms such as the modulation of immune cell infiltration. Elevated levels of this phosphatase is associated with the survival prognosis in some cancers [44]. In prostate cancer models, CDKN3 promotes cell proliferation and its knockdown inhibits cell growth, induces G1 phase cell cycle arrest, and increases apoptosis, inhibiting tumor growth. As an oncogenic factor, CDKN3 is overexpressed in prostate cancer tissues compared to normal prostate tissues, and its increased expression correlates with higher tumor stages and more advanced disease [45]. Collectively, the results of current AI align with other data [45,46], pointing to CDKN3’s oncogenic role in prostate cancer.

Anti-Müllerian hormone (AMH) is a peptide hormone from the TGF-β superfamily, a class of molecules regulating apoptosis, differentiation, and growth in many cells. AMH is produced mainly by Sertoli cells in the testes and plays a physiological role. Both in vitro and in vivo models have demonstrated that AMH inhibits proliferation and induces apoptosis of prostate cancer cells through mechanisms involving NF-kB signaling pathways [47]. NF-κB activation by AMH leads to cell cycle arrest, increasing the accumulation of prostate cancer cells in the G1 phase. Moreover, NF-κB activation upregulates IEX-1, which is an immediate early gene involved in regulating cell growth and survival [47]. AMH plays a complex and mostly inhibitory role in prostate cancer. In the radical prostatectomy cohort of patients, low serum levels of AMH were correlated with aggressive prostate cancer [48]; however, in another study, no association was found between serum AMH and prostate cancer risk [49].

The identification of a distinct yet overlapping molecular signature for the intermediate-risk (Gleason 7) group provides significant biological insight into the progression of prostate cancer. In our GNN-based model, the Medium-risk group shares key markers (ASPN and C2) with the High-risk group. The identification of a shared genetic signature between these groups may suggest that the biological events of aggressive cancer are already running before the tumor even looks high-grade under a microscope. Clinically, Gleason 7 represents a heterogeneous population where Grade Group 2 (3 + 4) may behave indolently, while Grade Group 3 (4 + 3) often mimics high-risk disease [50]. Our AI-driven classification suggests that the Medium group is not merely a middle ground, but a molecular bridge. This discovery brings a much-needed layer of clarity to clinical decision-making. If we see these bridge markers (ASPN, C2) in a patient who looks medium-risk, it tells us that their cancer is likely on a fast track toward high-grade aggressiveness. These patients might benefit from radical intervention sooner rather than later. On the other hand, if these markers are missing, it gives both the doctor and the patient the confidence to choose a more conservative path, sparing them the life-altering side effects of unnecessary treatment. By looking past static images and into the dynamic network of mRNA, our model moves beyond static histology toward a dynamic assessment of tumor biological potential, offering a pathway toward more personalized urologic oncology.

The TNMD gene (tenomodulin) is primarily known as a tendon-specific marker important for tendon maturation, stem/progenitor cell regulation, and anti-angiogenic functions. Pan-cancer analysis found significant associations between TNMD and immune-related pathways, indicating its possible role in the tumor microenvironment or immune modulation in different cancers [51].

The VWA5B2 gene encodes the von Willebrand factor A domain-containing protein 5B2. In analyses using The Cancer Genome Atlas prostate cancer dataset, VWA5B2 expression was found to be significantly associated with biochemical recurrence risk in prostate cancer after radical prostatectomy. The model using seven identified genes showed good predictive accuracy for biochemical recurrence risk-free probability at 1, 3, and 5 years, with AUC values ranging from 77% to 86% [52].

Androgen receptor (AR) signaling is essential for the growth and progression of prostate cancer and is regulated by cytochrome P450 3A5 (CYP3A5) [53,54]. CYP3A5 controls the functioning of the AR by helping its nuclear translocation and testosterone metabolism. CYP3A5 is highly expressed in normal prostate basolateral cells but is reduced or absent in tumor tissue [55]. The CYP3A5*3 polymorphism may influence the enzyme activity and be linked to increased prostate cancer risk [56], suggesting its loss may remove a protective metabolic function in regulating local androgen levels

ST6GALNAC5 is part of the ST6GALNAC family of sialyltransferases, which play a role in cancer progression and metastasis by altering the sialylation of cell surface glycoproteins. ST6GALNAC5 is associated with prostate cancer progression and metastasis. Overexpression of ST6GALNAC5 correlates with higher Gleason score and perineural invasion, and mechanistically enhances cancer cell proliferation and invasion in preclinical prostate models [57]. GATA2 is a transcription factor that upregulates ST6GALNAC5 expression and promotes prostate cell invasion. It is reported that miR-182 contributed to the invasion and proliferation of prostate cancer cells by targeting ST6GALNAC5 expression [58].

This study has several limitations. First, while the integration of transcriptomic data and functional networks identifies biologically plausible candidates, this study remains purely computational. In the absence of direct functional validation such as in vitro knockdown or overexpression experiments, the identified gene signatures should be interpreted as strong associations rather than confirmed mechanistic drivers of prostate cancer aggressiveness. Functional validation of these genes in clinical cohorts will help confirm their roles as prognostic biomarkers or therapeutic targets. Second, although we employed a severe data-splitting strategy (70% training, 10% validation, and 20% testing) and ensured that feature selection was performed strictly within the training set to prevent data leakage, the generalizability of our findings remains to be established. Finally, the clinical utility of this framework as a decision-making tool has yet to be proven. Future research incorporating independent, multi-center clinical cohorts and prospective datasets will be essential to confirm the robustness of our model. Moreover, integrating mRNA profiles with additional layers of biological data (proteomics or epigenomics) may further refine risk prediction and facilitate the translation of these computational insights into routine urologic oncology practice. Future studies integrating mRNA profiles with additional molecular and clinical data, as well as external validation in larger cohorts, will be essential to refine risk prediction and improve translation into clinical practice.

## Conclusion

The proposed model effectively distinguished between low-, medium-, and high-risk patient groups as indicated by Gleason scores by integrating feature selection methods with a GNN classifier. With an overall test accuracy of 80% and AUC values of 0.87, 0.86, and 0.95 for each of the three risk categories; respectively, the model demonstrated strong predictive performance.

Based on the AI analysis of dysregulated mRNA levels in prostate cancer, the observed distinct gene expression patterns provide essential insights into molecular changes linked to tumor aggressiveness and progression. The High Gleason Score group shows dysregulation of genes such as ASPN, GMNN, PEBP4, C2, and KNCK17. These genes are likely involved in pathways that promote tumor progression, invasion, and poor prognosis. For example, ASPN has been implicated in extracellular matrix remodeling and facilitation of tumor invasion, while GMNN regulates cell cycle progression and DNA replication licensing, supporting aggressive tumor cell proliferation. The presence of C2 suggests involvement of immune and inflammatory pathways, which are increasingly recognized as critical players in advanced prostate cancer biology. The Medium group’s top genes (C2, IGSF1, ASPN, CDKN3, AMH) include a mixture of genes contributing to immune regulation (C2), cell growth control (CDKN3), cell adhesion or signaling (IGSF1), stromal interaction (ASPN), and potentially tumor suppression (AMH). This pattern reflects a complex balance between tumor-promoting and inhibitory signals in tumors of intermediate aggressiveness, suggesting these genes could serve as early markers for progression or targets for intervention before the tumor becomes high-risk. Dysregulated genes in the Low group (TNMD, VWA5B2, ST6GALNAC5, CYP3A5, PHGR1) differ from those in higher groups and may reflect slower-growing, less aggressive tumors or different cellular pathways. Notably, CYP3A5 is a cytochrome P450 enzyme implicated in drug metabolism and steroid hormone synthesis, which might influence tumor biology in low-grade cancers. Based on the results, the High and Medium groups share some overlapping genes (like ASPN and C2), indicating a continuum of molecular changes from intermediate to high-grade tumors. In contrast, the low group shows a unique gene expression signature, marking tumors with less aggressive biology. This molecular grouping can present important diagnostic, prognostic, and therapeutic implications.

## Supporting information

S1 File (RAR)

S2 File (RAR)

S3 File (RAR)

S4 File (RAR)
