# Supplementary material for: Graph neural network-based risk stratification of prostate cancer using gene expression and SHAP interpretability
Source: PLoS One. 2026 Sep 8;21(9):e0356727. doi: 10.1371/journal.pone.0356727 (PMC13552954; doi:10.1371/journal.pone.0356727)
Supplement: Supplementary file 1 [file pone.0356727.s005.docx]

**Dear editorial team,**

Thank you for the opportunity to revise our manuscript. We addressed the issues.

1. Please upload a Response to Reviewers letter which should include a point by point response to each of the points made by the Editor and / or Reviewers. (This should be uploaded as a 'Response to Reviewers' file type.)

**Reply.** We uploaded a separate document titled "Response to Reviewers," which provides a comprehensive, point-by-point response to all comments made by the Editor and Reviewers.

2. Kindly update your main document removing highlighted text and using only black font color.

**Reply.** The main manuscript updated to comply with the journal’s formatting requirements. All highlighted text has been removed, and the font color has been set to black throughout the entire document.

3. Please note that funding information should not appear in the Acknowledgments section or other areas of your manuscript. We will only publish funding information present in the Funding Statement section of the online submission form. Please remove any funding-related text from the manuscript.

**Reply.** As requested, we have removed all funding-related information and financial acknowledgments from the Acknowledgments section and all other parts of the manuscript.

4. We are unable to open your Supporting Information files

**Reply.** We would like to clarify that all statistical analyses in this study were performed using Python. Accordingly, the outputs have been uploaded as “.pkl” (Pickle) files format to preserve the integrity of the data structures and analytical outputs. These files contain all the processed data required for the generation of all tables and figures presented in the manuscript.
