# Supplementary material for: Graph neural network-based risk stratification of prostate cancer using gene expression and SHAP interpretability
Source: PLoS One. 2026 Sep 8;21(9):e0356727. doi: 10.1371/journal.pone.0356727 (PMC13552954; doi:10.1371/journal.pone.0356727)
Supplement: Peer review history

# Peer review: Decision Letter 0

**DOI:** 10.1371/journal.pone.0356727.r001

**Authors:** Jan Philipp Radtke

1 Apr 2026

Dear Dr. Vahed,

thank you for submitting your manuscript to PLOS ONE. After careful consideration, we feel that it has merit but does not fully meet PLOS ONE’s publication criteria as it currently stands. Therefore, we invite you to submit a revised version of the manuscript that addresses the points raised during the review process.

Please submit your revised manuscript by May 16 2026 11:59PM. If you will need more time than this to complete your revisions, please reply to this message or contact the journal office at plosone@plos.org. When you're ready to submit your revision, log on to https://www.editorialmanager.com/pone/ and select the 'Submissions Needing Revision' folder to locate your manuscript file.

- A letter that responds to each point raised by the academic editor and reviewer(s). You should upload this letter as a separate file labeled 'Response to Reviewers'.
- A marked-up copy of your manuscript that highlights changes made to the original version. You should upload this as a separate file labeled 'Revised Manuscript with Track Changes'.
- An unmarked version of your revised paper without tracked changes. You should upload this as a separate file labeled 'Manuscript'.

If you would like to make changes to your financial disclosure, please include your updated statement in your cover letter. Guidelines for resubmitting your figure files are available below the reviewer comments at the end of this letter.

If applicable, we recommend that you deposit your laboratory protocols in protocols.io to enhance the reproducibility of your results. Protocols.io assigns your protocol its own identifier (DOI) so that it can be cited independently in the future. For instructions see: https://journals.plos.org/plosone/s/submission-guidelines#loc-laboratory-protocols. Additionally, PLOS ONE offers an option for publishing peer-reviewed Lab Protocol articles, which describe protocols hosted on protocols.io. Read more information on sharing protocols at https://plos.org/protocols?utm_medium=editorial-email&utm_source=authorletters&utm_campaign=protocols.

As the corresponding author, your ORCID iD is verified in the submission system and will appear in the published article. PLOS supports the use of ORCID, and we encourage all coauthors to register for an ORCID iD and use it as well. Please encourage your coauthors to verify their ORCID iD within the submission system before final acceptance, as unverified ORCID iDs will not appear in the published article. *Only*  the individual author can complete the verification step; PLOS staff *cannot*  verify ORCID iDs on behalf of authors.

We look forward to receiving your revised manuscript.

Kind regards,

Jan Philipp Radtke, MBA

Academic Editor

PLOS One

Journal Requirements:

When submitting your revision, we need you to address these additional requirements.

1. Please ensure that your manuscript meets PLOS ONE's style requirements, including those for file naming. The PLOS ONE style templates can be found at

https://journals.plos.org/plosone/s/file?id=wjVg/PLOSOne_formatting_sample_main_body.pdf and

https://journals.plos.org/plosone/s/file?id=ba62/PLOSOne_formatting_sample_title_authors_affiliations.pdf

2. Please note that PLOS One has specific guidelines on code sharing for submissions in which author-generated code underpins the findings in the manuscript. In these cases, we expect all author-generated code to be made available without restrictions upon publication of the work. Please review our guidelines at https://journals.plos.org/plosone/s/materials-and-software-sharing#loc-sharing-code and ensure that your code is shared in a way that follows best practice and facilitates reproducibility and reuse.

3. We note that the grant information you provided in the ‘Funding Information’ and ‘Financial Disclosure’ sections do not match.

When you resubmit, please ensure that you provide the correct grant numbers for the awards you received for your study in the ‘Funding Information’ section.

4. Thank you for stating the following financial disclosure:

“The author(s) received no specific funding for this work”

At this time, please address the following queries:

a) Please clarify the sources of funding (financial or material support) for your study. List the grants or organizations that supported your study, including funding received from your institution.

b) State what role the funders took in the study. If the funders had no role in your study, please state: “The funders had no role in study design, data collection and analysis, decision to publish, or preparation of the manuscript.”

c) If any authors received a salary from any of your funders, please state which authors and which funders.

d) If you did not receive any funding for this study, please state: “The authors received no specific funding for this work.”

Please include your amended statements within your cover letter; we will change the online submission form on your behalf.

5. We note that your Data Availability Statement is currently as follows: All relevant data are within the manuscript and its Supporting Information files.

Please confirm at this time whether or not your submission contains all raw data required to replicate the results of your study. Authors must share the “minimal data set” for their submission. PLOS defines the minimal data set to consist of the data required to replicate all study findings reported in the article, as well as related metadata and methods (https://journals.plos.org/plosone/s/data-availability#loc-minimal-data-set-definition).

For example, authors should submit the following data:

- The values behind the means, standard deviations and other measures reported;

- The values used to build graphs;

- The points extracted from images for analysis.

Authors do not need to submit their entire data set if only a portion of the data was used in the reported study.

If your submission does not contain these data, please either upload them as Supporting Information files or deposit them to a stable, public repository and provide us with the relevant URLs, DOIs, or accession numbers. For a list of recommended repositories, please see https://journals.plos.org/plosone/s/recommended-repositories.

If there are ethical or legal restrictions on sharing a de-identified data set, please explain them in detail (e.g., data contain potentially sensitive information, data are owned by a third-party organization, etc.) and who has imposed them (e.g., an ethics committee). Please also provide contact information for a data access committee, ethics committee, or other institutional body to which data requests may be sent. If data are owned by a third party, please indicate how others may request data access.

6. If the reviewer comments include a recommendation to cite specific previously published works, please review and evaluate these publications to determine whether they are relevant and should be cited. There is no requirement to cite these works unless the editor has indicated otherwise.

[Note: HTML markup is below. Please do not edit.]

Reviewers' comments:

Reviewer's Responses to Questions

**Comments to the Author**

1. Is the manuscript technically sound, and do the data support the conclusions?

Reviewer #1: Yes

Reviewer #2: Partly

**********

2. Has the statistical analysis been performed appropriately and rigorously?

Reviewer #1: Yes

Reviewer #2: N/A

**********

3. Have the authors made all data underlying the findings in their manuscript fully available?

The PLOS Data policy

Reviewer #1: No

Reviewer #2: Yes

**********

4. Is the manuscript presented in an intelligible fashion and written in standard English?

Reviewer #1: Yes

Reviewer #2: Yes

**********

Reviewer #1: Graph Neural Network-Based Risk Stratification of Prostate Cancer Using Gene Expression and SHAP Interpretability

This manuscript presents a compelling application of Graph Neural Networks (GNNs) for prostate cancer classification by integrating gene expression data with functional interaction networks. However, several critical methodological concerns must be addressed to ensure the validity and reproducibility of the findings. The lack of a specified code repository and formal TCGA citations hinders transparency. Furthermore, the data preprocessing pipeline requires clarification, specifically regarding the normalization of TCGA count matrices and the unconventional "impute-then-filter" sequence for missing values. Given the absence of an independent validation cohort, the current 80/20 hold-out strategy is insufficient to rule out overfitting; the authors should instead implement a k-fold cross-validation approach to demonstrate the stability of their feature selection. While the use of SMOTE addresses class imbalance, the reliance on AUROC may mask a high false-positive rate. The inclusion of AUPRC metrics is essential to confirm that the model maintains high precision. Additionally, the dual-component architecture of the GNN—balancing the gene network and expression data—requires a more detailed description.

General Feedback

- Biomarker Validation: A significant strength of this work is the identification of biologically relevant biomarkers. The use of SHAP for explainability is effective, and the results correlate well with established biomarkers from the TCGA dataset in the current literature.

- Model Innovation: The application of Graph Neural Networks (GNN) to integrate gene expression with functional interaction networks is a compelling approach.

Major Methodological Concerns

- Validation Strategy: In the absence of an independent validation cohort, the current 80/20 hold-out strategy is insufficient. A k-fold cross-validation approach should be implemented to ensure that the feature selection and model performance are not artifacts of a single random split.

- Data Preprocessing & Imputation: The authors must clarify the TCGA normalization pipeline. Furthermore, the "impute-then-filter" sequence for missing values is unconventional; standard practice is to remove features with high missingness before performing imputation. The specific imputation method must be stated.

- Class Imbalance (SMOTE): While SMOTE addresses the unbalanced dataset, the reliance on AUROC may mask an increase in false positives. The authors should include AUPRC (Precision-Recall) metrics to confirm that precision is maintained.

- Comparative Analysis: The comparison with other predictors is currently lacking. The authors should specify the SVM kernel used (linear vs. non-linear) and include a comparison with simple linear models to justify whether the GNN’s complexity actually provides a significant performance gain.

- Database Justification: The authors should justify the selection of GeneMANIA over more widely recognized protein-protein interaction databases like STRINGdb.

Technical & Minor Comments

- Network Architecture: A detailed description of the GNN is required, including the number of layers, number of neurons per layer, and the specific process used for hyperparameter tuning.

- Code & Citations: A link to the code repository should be provided to ensure reproducibility, and formal citations for the TCGA dataset are required.

- Data Visualization: In all PCA plots, numerical class labels (0, 1, 2, 3) should be replaced with descriptive names (e.g., High, Mid, Low) to improve readability.

- Architecture Description: The dual-component architecture (modeling the gene network vs. gene expression) requires a more detailed explanation in the text to help the reader understand the data flow.

Reviewer #2: Peer Review Report

General Comments

This manuscript presents the development of an artificial intelligence–based model for risk stratification of prostate cancer using mRNA expression data, employing a graph neural network (GNN) architecture combined with SHAP-based interpretability.

The research question is clinically relevant. Accurate risk stratification remains central to treatment decision-making and the avoidance of overtreatment in prostate cancer. The concept of leveraging molecular data to estimate tumor aggressiveness aligns well with current trends in precision oncology.

The study demonstrates several strengths, including an innovative methodological framework and the identification of biologically plausible gene signatures. However, in its current form, the work remains largely exploratory. Key aspects—particularly external validation, clinical applicability, and methodological transparency—require further clarification and expansion.

Overall, the manuscript represents an interesting contribution but requires substantial revision before it can be considered for publication.

Major Comments

1. Lack of external validation

The model was developed and evaluated exclusively on a TCGA-based dataset. While an internal split into training, validation, and test cohorts is provided, it remains unclear to what extent the results generalize to independent patient populations.

Given the high dimensionality of gene expression data, there is a risk that the model captures dataset-specific patterns rather than robust biological signals. The high performance reported in the training set compared to the test set further highlights this concern.

Recommendation:

The authors should explicitly acknowledge the lack of external validation as a key limitation and discuss the generalizability of their findings more cautiously.

2. Limited clinical applicability

The model is based solely on mRNA expression data and does not incorporate established clinical variables such as PSA, tumor stage, imaging findings, or biopsy characteristics.

Since clinical risk stratification in prostate cancer is inherently multimodal, the practical added value of the proposed model remains unclear.

Recommendation:

The authors should better contextualize the potential clinical role of their model and clarify whether it is intended as an exploratory molecular approach or as a complementary tool to existing clinical frameworks.

3. Unclear clinical use scenario

The data used in this study are derived from publicly available datasets and do not directly reflect real-world clinical decision-making scenarios (e.g., pre-treatment or pre-biopsy settings).

As a result, the model effectively predicts Gleason-based risk groups from already well-characterized tumor tissue, rather than addressing a situation of diagnostic uncertainty.

In this context, the conclusion regarding “non-invasive risk stratification” appears overstated.

Recommendation:

The authors should clarify the intended use case and more explicitly discuss the limitations regarding real-world clinical application.

4. Limited transparency of the analysis pipeline

While the overall analytical framework is interesting, several aspects of the pipeline are insufficiently described, limiting reproducibility.

In particular, it remains unclear:

how the final set of selected genes was determined,

how the graph structure was constructed, and

how the different analytical steps were integrated.

Recommendation:

A clearer and more structured description of the methodology is needed, ideally outlining the pipeline step by step to improve reproducibility.

5. Insufficient discussion of the intermediate-risk group

The intermediate-risk group (Gleason 7) represents a clinically heterogeneous and decision-critical category.

Although the model identifies specific gene signatures for this group, their interpretation remains relatively superficial. A more in-depth discussion of the biological and potential clinical implications would strengthen the manuscript.

Recommendation:

The authors should expand on the findings related to the intermediate-risk group and better contextualize their relevance for clinical decision-making.

6. Overinterpretation of biological findings

Several of the identified genes are biologically plausible and have been previously associated with prostate cancer progression. However, the current study is based solely on computational analysis without functional validation.

Some conclusions regarding biological mechanisms and therapeutic implications therefore appear somewhat overstated.

Recommendation:

The authors should adopt a more cautious tone and clearly distinguish between associative findings and validated biological mechanisms.

Minor Comments

1. Methodological clarity

The description of the model and analytical steps should be further refined to improve clarity and reproducibility.

2. Figures

The SHAP and PCA figures contain relevant information but are not always easy to interpret.

Recommendation:

Improving figure clarity and labeling would enhance readability.

3. Statistical reporting

Model performance is primarily reported using accuracy and AUC.

Recommendation:

Providing additional context regarding the stability of the results would strengthen the analysis.

4. Language and terminology

The manuscript is generally well written, but minor linguistic revisions and more consistent terminology would improve readability.

Strengths

Innovative application of graph neural networks

Integration of biological network information

Use of interpretable AI methods (SHAP)

Identification of biologically plausible gene signatures

Focus on a clinically relevant problem

Conclusion

This study demonstrates that AI-based analysis of mRNA expression data has the potential to distinguish between prostate cancer risk groups and to identify relevant molecular patterns associated with tumor aggressiveness.

However, the current work remains exploratory due to the lack of external validation, limited clinical integration, and insufficient methodological transparency.

With appropriate revisions, the manuscript could make a valuable contribution to the field of AI-driven molecular oncology.

Recommendation

Major Revision

**********

what does this mean?). If published, this will include your full peer review and any attached files.

If you choose “no”, your identity will remain anonymous but your review may still be made public.

**Do you want your identity to be public for this peer review?**  For information about this choice, including consent withdrawal, please see our Privacy Policy

Reviewer #1: No

Reviewer #2: No

**********

[NOTE: If reviewer comments were submitted as an attachment file, they will be attached to this email and accessible via the submission site. Please log into your account, locate the manuscript record, and check for the action link "View Attachments". If this link does not appear, there are no attachment files.]

To ensure your figures meet our technical requirements, please review our figure guidelines: https://journals.plos.org/plosone/s/figures

You may also use PLOS’s free figure tool, NAAS, to help you prepare publication quality figures: https://journals.plos.org/plosone/s/figures#loc-tools-for-figure-preparation.

NAAS will assess whether your figures meet our technical requirements by comparing each figure against our figure specifications.

# Author comment: Author response to Decision Letter 1

**DOI:** 10.1371/journal.pone.0356727.r002

26 May 2026

Review Comments to the Author

Reviewer #1: Peer Review Report

This manuscript presents a compelling application of Graph Neural Networks (GNNs) for prostate cancer classification by integrating gene expression data with functional interaction networks. However, several critical methodological concerns must be addressed to ensure the validity and reproducibility of the findings. The lack of a specified code repository and formal TCGA citations hinders transparency. Furthermore, the data preprocessing pipeline requires clarification, specifically regarding the normalization of TCGA count matrices and the unconventional "impute-then-filter" sequence for missing values. Given the absence of an independent validation cohort, the current 80/20 hold-out strategy is insufficient to rule out overfitting; the authors should instead implement a k-fold cross-validation approach to demonstrate the stability of their feature selection. While the use of SMOTE addresses class imbalance, the reliance on AUROC may mask a high false-positive rate. The inclusion of AUPRC metrics is essential to confirm that the model maintains high precision. Additionally, the dual-component architecture of the GNN—balancing the gene network and expression data—requires a more detailed description.

General Feedback

- Biomarker Validation: A significant strength of this work is the identification of biologically relevant biomarkers. The use of SHAP for explainability is effective, and the results correlate well with established biomarkers from the TCGA dataset in the current literature.

- Model Innovation: The application of Graph Neural Networks (GNN) to integrate gene expression with functional interaction networks is a compelling approach.

Author response: We sincerely thank the reviewer for their thorough and insightful evaluation of our manuscript. We appreciate the detailed methodological feedback, as well as the recognition of the strengths of our work, including the biological relevance of the identified biomarkers, the utility of SHAP based explainability, and the innovation of integrating gene expression with functional interaction networks through a GNN framework. The reviewer’s comments are extremely helpful in improving the clarity, rigor, and reproducibility of the study. We have carefully addressed each point, including clarifying the preprocessing pipeline, strengthening the model description, incorporating additional performance metrics, and explicitly acknowledging limitations such as the lack of external validation. We are grateful for the reviewer’s constructive guidance, which has substantially enhanced the quality of the revised manuscript.

Major Methodological Concerns

- Validation Strategy: In the absence of an independent validation cohort, the current 80/20 hold-out strategy is insufficient. A k-fold cross-validation approach should be implemented to ensure that the feature selection and model performance are not artifacts of a single random split.

Author response: We thank the reviewer for this important comment. We agree that validation strategy is critical, particularly in high-dimensional genomic studies. In our framework, we employed a predefined data partitioning strategy consisting of 70% training, 10% validation, and 20% independent test data. The final model performance reported in the manuscript is based exclusively on the held out test set, which was not used during model training or hyperparameter optimization.

We acknowledge that k fold cross validation is a commonly used strategy to further assess robustness. However, given the computational complexity of the graph neural network model and the relatively limited cohort size, we opted for a strict hold out design with an independent test set to minimize information leakage and preserve a fully unseen evaluation subset. Importantly, feature selection was performed exclusively within the training data to avoid optimistic bias. The consistency of performance between validation and test sets suggests that the model does not rely on artifacts of a single random split. Nevertheless, we agree that the absence of an external independent cohort remains a limitation, and we now explicitly state this in the Discussion. Future studies incorporating multi-cohort validation or prospective datasets will be essential to further establish generalizability.

- Data Preprocessing & Imputation: The authors must clarify the TCGA normalization pipeline. Furthermore, the "impute-then-filter" sequence for missing values is unconventional; standard practice is to remove features with high missingness before performing imputation. The specific imputation method must be stated.

Author response: Thank you for highlighting this point. We have revised the Methods section to clarify the preprocessing pipeline applied to the TCGA gene expression data. In the dataset used in this study, the TCGA mRNA expression matrix did not contain missing values; therefore, no imputation procedure was required. Consequently, the concern regarding the order of imputation and filtering does not apply in this case.

To improve transparency and reproducibility, we have expanded the description of the preprocessing steps in the revised manuscript, including how the gene expression matrix was organized and prepared prior to feature selection and model training. These steps now clearly describe the preparation of the expression matrix, normalization procedure, and subsequent feature selection using ANOVA and ReliefF before the classification stage.

We appreciate the reviewer’s suggestion, which helped us improve the clarity of the preprocessing description in the manuscript.

- Class Imbalance (SMOTE): While SMOTE addresses the unbalanced dataset, the reliance on AUROC may mask an increase in false positives. The authors should include AUPRC (Precision-Recall) metrics to confirm that precision is maintained.

Author response: We agree with the reviewer that AUROC alone may not fully capture model behavior under class imbalance and may obscure increases in false positives, particularly when oversampling techniques such as SMOTE are applied. To address this concern, we have expanded the evaluation framework in the revised manuscript to include precision–recall–based metrics.

Specifically, we now report per class precision, recall, and F1 score using a one vs rest strategy, which allows a more granular assessment of false positive and false negative trade offs for each risk group. In addition, we include area under the precision–recall curve (AUPRC) where applicable, providing a threshold independent measure that is more informative for imbalanced classification settings.

These additional metrics complement AUROC and accuracy by explicitly quantifying whether precision is preserved after applying SMOTE, thereby offering a more comprehensive and transparent evaluation of model performance under class imbalance. We thank the reviewer for this valuable suggestion, which has strengthened the rigor of the performance analysis.

- Comparative Analysis: The comparison with other predictors is currently lacking. The authors should specify the SVM kernel used (linear vs. non-linear) and include a comparison with simple linear models to justify whether the GNN’s complexity actually provides a significant performance gain.

Author response: We appreciate this important suggestion. We have clarified the baseline models used for comparison and specified the SVM configuration in the revised manuscript. We also expanded the comparative analysis to include simpler models, allowing a more direct assessment of whether the graph neural network provides measurable benefit beyond conventional linear classifiers. This revision helps better justify the added complexity of the proposed model.

- Database Justification: The authors should justify the selection of GeneMANIA over more widely recognized protein-protein interaction databases like STRINGdb.

Author response: Thank you for this comment. We have added a justification for using GeneMANIA in the revised manuscript. Our choice was motivated by its ability to provide functionally relevant gene-gene interaction information and its suitability for constructing biologically informed networks for downstream graph-based learning. We now discuss this choice more explicitly and acknowledge that alternative databases such as STRINGdb could also be considered in future studies.

Technical & Minor Comments

- Network Architecture: A detailed description of the GNN is required, including the number of layers, number of neurons per layer, and the specific process used for hyperparameter tuning.

Author response: We agree that methodological transparency is essential. We have expanded the Methods section to provide a clearer description of the graph neural network architecture, including the main design components of the model and the strategy used for hyperparameter optimization. Where possible, we have added the number of layers, layer sizes, and tuning procedure to improve reproducibility.

- Code & Citations: A link to the code repository should be provided to ensure reproducibility, and formal citations for the TCGA dataset are required.

Author response: We appreciate this comment. We have revised the manuscript to include the appropriate TCGA citations and have added a statement regarding code availability / repository access where applicable. These changes are intended to improve reproducibility and ensure proper attribution of the source data.

- Data Visualization: In all PCA plots, numerical class labels (0, 1, 2, 3) should be replaced with descriptive names (e.g., High, Mid, Low) to improve readability.

Author response: Thank you. We have updated the figures captions so that class labels are shown using descriptive group names rather than numeric codes, which improves readability and interpretability.

- Architecture Description: The dual-component architecture (modeling the gene network vs. gene expression) requires a more detailed explanation in the text to help the reader understand the data flow.

Author response: We agree and have revised the manuscript to explain the architecture more clearly. The updated description now walks the reader through the data flow from gene expression and network construction to graph-based classification and SHAP interpretation, making the dual-component design easier to follow.

Reviewer #2: Peer Review Report

General Comments

This manuscript presents the development of an artificial intelligence–based model for risk stratification of prostate cancer using mRNA expression data, employing a graph neural network (GNN) architecture combined with SHAP-based interpretability. The research question is clinically relevant. Accurate risk stratification remains central to treatment decision-making and the avoidance of overtreatment in prostate cancer. The concept of leveraging molecular data to estimate tumor aggressiveness aligns well with current trends in precision oncology. The study demonstrates several strengths, including an innovative methodological framework and the identification of biologically plausible gene signatures. However, in its current form, the work remains largely exploratory. Key aspects—particularly external validation, clinical applicability, and methodological transparency—require further clarification and expansion. Overall, the manuscript represents an interesting contribution but requires substantial revision before it can be considered for publication.

Author response: We sincerely thank the reviewer for their thoughtful and constructive evaluation of our work. We particularly appreciate the recognition of the clinical relevance of prostate cancer risk stratification, the innovative nature of our GNN based framework combined with SHAP interpretability, and the identification of biologically plausible gene signatures. We fully agree that, in its current form, the study should be regarded as exploratory, and we have revised the manuscript to more clearly reflect this. In response to the reviewer’s comments, we have expanded our discussion of methodological transparency, clarified the clinical context and potential applicability of the model, and more explicitly acknowledged the lack of external validation as a key limitation. We are grateful for these insights, which have guided substantial revisions and, we believe, have significantly strengthened the manuscript.

Major Comments

1.Lack of external validation

The model was developed and evaluated exclusively on a TCGA-based dataset. While an internal split into training, validation, and test cohorts is provided, it remains unclear to what extent the results generalize to independent patient populations. Given the high dimensionality of gene expression data, there is a risk that the model captures dataset-specific patterns rather than robust biological signals. The high performance reported in the training set compared to the test set further highlights this concern.

Recommendation:

The authors should explicitly acknowledge the lack of external validation as a key limitation and discuss the generalizability of their findings more cautiously.

Author response: We agree. We have strengthened the Discussion to clearly acknowledge that the model has not yet been validated on an independent external cohort. We now present the findings as exploratory and emphasize that the results should be interpreted cautiously until replicated in external datasets.

We agree that validation strategy is critical, particularly in high-dimensional genomic studies. In our framework, we employed a predefined data partitioning strategy consisting of 70% training, 10% validation, and 20% independent test data. The final model performance reported in the manuscript is based exclusively on the held out test set, which was not used during model training or hyperparameter optimization.

We acknowledge that k fold cross validation is a commonly used strategy to further assess robustness. However, given the computational complexity of the graph neural network model and the relatively limited cohort size, we opted for a strict hold out design with an independent test set to minimize information leakage and preserve a fully unseen evaluation subset.

Importantly, feature selection was performed exclusively within the training data to avoid optimistic bias. The consistency of performance between validation and test sets suggests that the model does not rely on artifacts of a single random split.

Nevertheless, we agree that the absence of an external independent cohort remains a limitation, and we now explicitly state this in the Discussion. Future studies incorporating multi-cohort validation or prospective datasets will be essential to further establish generalizability.

2. Limited clinical applicability

The model is based solely on mRNA expression data and does not incorporate established clinical variables such as PSA, tumor stage, imaging findings, or biopsy characteristics. Since clinical risk stratification in prostate cancer is inherently multimodal, the practical added value of the proposed model remains unclear.

Recommendation:

The authors should better contextualize the potential clinical role of their model and clarify whether it is intended as an exploratory molecular approach or as a complementary tool to existing clinical frameworks.

Author response: Thank you for this important observation. We have revised the manuscript to better position the study as a molecular and computational proof-of-concept rather than a standalone clinical decision tool. We now clarify that the model is intended as a complementary approach that may eventually be integrated with clinical variables in future multimodal frameworks.

3. Unclear clinical use scenario

The data used in this study are derived from publicly available datasets and do not directly reflect real-world clinical decision-making scenarios (e.g., pre-treatment or pre-biopsy settings). As a result, the model effectively predicts Gleason-based risk groups from already well-characterized tumor tissue, rather than addressing a situation of diagnostic uncertainty. In this context, the conclusion regarding “non-invasive risk stratification” appears overstated.

Recommendation

Attachment Submitted filename: Response to Reviewers.docx

# Peer review: Decision Letter 1

**DOI:** 10.1371/journal.pone.0356727.r003

**Authors:** Stanisław Jacek Wroński

6 Aug 2026

Graph Neural Network-Based Risk Stratification of Prostate Cancer Using Gene Expression and SHAP Interpretability

PONE-D-26-08240R1

Dear Dr. Sepideh Vahed,

we’re pleased to inform you that your manuscript has been judged scientifically suitable for publication and will be formally accepted for publication once it meets all outstanding technical requirements.

Within one week, you’ll receive an e-mail detailing the required amendments. When these have been addressed, you’ll receive a formal acceptance letter and your manuscript will be scheduled for publication.

An invoice will be generated when your article is formally accepted. Please note, if your institution has a publishing partnership with PLOS and your article meets the relevant criteria, all or part of your publication costs will be covered. Please make sure your user information is up-to-date by logging into Editorial Manager at Editorial Manager® and clicking the ‘Update My Information' link at the top of the page. For questions related to billing, please contact billing support.

If your institution or institutions have a press office, please notify them about your upcoming paper to help maximize its impact. If they’ll be preparing press materials, please inform our press team as soon as possible -- no later than 48 hours after receiving the formal acceptance. Your manuscript will remain under strict press embargo until 2 pm Eastern Time on the date of publication. For more information, please contact onepress@plos.org.

Kind regards,

Stanisław Jacek Wroński, M.D., Ph.D, FEBU

Academic Editor

PLOS One

Reviewer's Responses to Questions

**Comments to the Author**

Reviewer #1: All comments have been addressed

**********

2. Is the manuscript technically sound, and do the data support the conclusions?

Reviewer #1: Yes

**********

3. Has the statistical analysis been performed appropriately and rigorously?

Reviewer #1: Yes

**********

4. Have the authors made all data underlying the findings in their manuscript fully available?

The PLOS Data policy

Reviewer #1: Yes

**********

5. Is the manuscript presented in an intelligible fashion and written in standard English?

Reviewer #1: Yes

**********

Reviewer #1: The author have replies to all my comments and change the the manuscript accordingly.

Although the limitation of not having an external / independent validation cohort is still huge limitation, the methodology presented deserve to be published.

Minor : The comment about the PCA plots was not fully fixed, please change the label in plots with High , Median and Low . The class label as number are too hard to read.

**********

what does this mean?). If published, this will include your full peer review and any attached files.

If you choose “no”, your identity will remain anonymous but your review may still be made public.

**Do you want your identity to be public for this peer review?**  For information about this choice, including consent withdrawal, please see our Privacy Policy

Reviewer #1: No

**********

# Editor report: Acceptance letter

**DOI:** 10.1371/journal.pone.0356727.r004

**Authors:** Stanisław Jacek Wroński

PONE-D-26-08240R1

PLOS One

Dear Dr. Zununi Vahed,

I'm pleased to inform you that your manuscript has been deemed suitable for publication in PLOS One. Congratulations! Your manuscript is now being handed over to our production team.

At this stage, our production department will prepare your paper for publication. This includes ensuring the following:

* All references, tables, and figures are properly cited

* All relevant supporting information is included in the manuscript submission,

* There are no issues that prevent the paper from being properly typeset

You will receive further instructions from the production team, including instructions on how to review your proof when it is ready. Please keep in mind that we are working through a large volume of accepted articles, so please give us a few days to review your paper and let you know the next and final steps.

Lastly, if your institution or institutions have a press office, please let them know about your upcoming paper now to help maximize its impact. If they'll be preparing press materials, please inform our press team within the next 48 hours. Your manuscript will remain under strict press embargo until 2 pm Eastern Time on the date of publication. For more information, please contact onepress@plos.org.

You will receive an invoice from PLOS for your publication fee after your manuscript has reached the completed accept phase. If you receive an email requesting payment before acceptance or for any other service, this may be a phishing scheme. Learn how to identify phishing emails and protect your accounts at https://explore.plos.org/phishing.

If we can help with anything else, please email us at customercare@plos.org.

Thank you for submitting your work to PLOS One and supporting open access.

Kind regards,

PLOS ONE Editorial Office Staff

on behalf of

Dr. Stanisław Jacek Wroński

Academic Editor

PLOS One
